# Stomatal conductance tracks soil-to-leaf hydraulic conductance in faba bean and maize during soil drying

**DOI:** 10.1093/plphys/kiac422

**Published:** 2022-09-13

**Authors:** Yannik Müllers, Johannes A Postma, Hendrik Poorter, Dagmar van Dusschoten

**Affiliations:** Plant Sciences (IBG-2), Forschungszentrum Jülich GmbH, D-52425 Jülich, Germany; Plant Sciences (IBG-2), Forschungszentrum Jülich GmbH, D-52425 Jülich, Germany; Plant Sciences (IBG-2), Forschungszentrum Jülich GmbH, D-52425 Jülich, Germany; Department of Biological Sciences, Macquarie University, North Ryde, NSW, 2109 Australia; Plant Sciences (IBG-2), Forschungszentrum Jülich GmbH, D-52425 Jülich, Germany

## Abstract

Although regulation of stomatal conductance is widely assumed to be the most important plant response to soil drying, the picture is incomplete when hydraulic conductance from soil to the leaf, upstream of the stomata, is not considered. Here, we investigated to what extent soil drying reduces the conductance between soil and leaf, whether this reduction differs between species, how it affects stomatal regulation, and where in the hydraulic pathway it occurs. To this end, we noninvasively and continuously measured the total root water uptake rate, soil water potential, leaf water potential, and stomatal conductance of 4-week-old, pot-grown maize (*Zea mays*) and faba bean (*Vicia faba*) plants during 4 days of water restriction. In both species, the soil–plant conductance, excluding stomatal conductance, declined exponentially with soil drying and was reduced to 50% above a soil water potential of −0.1 MPa, which is far from the permanent wilting point. This loss of conductance has immediate consequences for leaf water potential and the associated stomatal regulation. Both stomatal conductance and soil–plant conductance declined at a higher rate in faba bean than in maize. Estimations of the water potential at the root surface and an incomplete recovery 22 h after rewatering indicate that the loss of conductance, at least partly, occurred inside the plants, for example, through root suberization or altered aquaporin gene expression. Our findings suggest that differences in the stomatal sensitivity among plant species are partly explained by the sensitivity of root hydraulic conductance to soil drying.

## Teaser

The hydraulic conductance between soil and leaf decreases exponentially with decreasing soil water potential, at a species-specific rate related to the decline rate of stomatal conductance.

## Introduction

To describe plant responses to soil drying, Feddes et al. (1978) proposed the concept of a water stress curve which still is the basis for most current root water uptake (RWU) models (Dos Santos et al., 2017). In this approach, a reduction factor of the transpiration rate is related to the soil water potential (Ψ_soil_; see Table 1 for abbreviations). Within a specific range of reducing Ψ_soil_, the transpiration rate is sustained implying a steadily reduced plant water potential to compensate for the reduced soil water potential. Below a critical Ψ_soil_, the transpiration rate is linearly reduced due to a partial closure of stomata avoiding a too strong decrease of the plant water potential. At the permanent wilting point, usually assumed to be −1.5 MPa, transpiration ceases. The exact shape of such a water stress curve depends on the extent of stomatal closure at a given level of soil drying. Variation of this stomatal sensitivity among species led to the classification in iso- and anisohydric species (Tardieu and Simmoneau, 1998), which is used to explain varying plant responses to soil drying (Pou et al., 2012; Sade et al., 2012; Hochberg et al., 2013; Attia et al., 2015). However, characterizing the hydraulic response of plants to soil drying by stomatal sensitivity only comes with limitations as stomatal control and leaf water potential regulation are not necessarily related when compared across various species (Martínez-Vilalta and Garcia-Forner, 2017).

**Table 1:** Abbreviations used in the article

Term	Meaning	Unit
*g*_s_	Stomatal conductance	mol H_2_O m^−2^ s^−1^
*h*	Soil matric potential	cm
*K*_RL_	Hydraulic conductance between root and leaf	cm h^−1^ Mpa^−1^
*K*_sat_	Saturated soil hydraulic conductivity	cm s^−1^
*K*_SL_	Hydraulic conductance between soil and leaf	cm h^−1^ Mpa^−1^
*K*_SR_	Hydraulic conductance between bulk soil and root surface	cm h^−1^ Mpa^−1^
*K*_soil_	Soil hydraulic conductivity	cm d^−1^
*L*	Root length	m
*A*	Leaf area	cm^2^
*r*_0_	Root radius	cm
*r*_b_	Radial distance from the root center defining the start of the bulk soil	cm
RWU	RWU rate	mL h^−1^
SWaP	Soil water profiler	
*U*_P_	Plant-driven RWU distribution with depth	mL cm^−3^ h^−1^
${\hat{U}}_{P}$	Normalized plant-driven RWU distribution with depth	
*U*_S_	Soil driven RWU redistribution	mL cm^−3^ h^−1^
*U*_tot_	Total RWU rate	mL h^−1^
*Z_i_*	Depth of soil layer *i*	cm
*Α*	Inverse of the air entry pressure	cm^−1^
*θ*	Volumetric soil water content	mL cm^−3^
$\frac{\partial \theta }{\partial t}$	Soil water depletion rate	mL cm^−3^ h^−1^
*λ*	Rate constant of the exponential relation between K_SL_ or g_s_ and Ψ_seq_	Mpa^−1^
*λ*_b_	Dimensionless pore size index of the Brooks–Corey model	
*τ*	Brooks–Corey parameter with *τ* = −2–3 λ_b_	
*φ*	Matrix flux potential	cm^2^ s^−1^
Ψ_seq_	Equivalent water potential in the bulk soil	Mpa
Ψ_seq_50_	Equivalent soil water potential at which the conductance (K_SL_ or g_s_) was reduced to 50% of its initial value	Mpa
Ψ_soil_	Water potential in the bulk soil	Mpa
Ψ_sr_	Water potential at the soil–root interface	Mpa
Ψ_seq, sr_	Equivalent water potential at the soil–root interface	Mpa

One potential cause for these inconsistencies is that not only the stomatal conductance, but also the hydraulic conductance upstream toward the stomata, from soil to leaf (*K*_SL_) can be affected by soil drying. For each part of this pathway, namely leaves (Cochard, 2002; Ryu et al., 2016), stems (Cochard, 2006; Li et al., 2009), and the soil root system (Saliendra and Meinzer, 1989; Nobel and Cui, 1992; Sperry and Saliendra, 1994; Cochard et al., 1996; Bourbia et al., 2021), a loss of conductance at a reduced soil water potential could be demonstrated. Potential reasons are air gaps between soil and root (North and Nobel, 1997), altered root aquaporin gene expression (Vandeleur et al., 2009; Grondin et al., 2016), suberization of the root epi-, endo-, and exodermis (North and Nobel, 1991; Cruz et al., 1992), or xylem embolisms (Cochard, 2006; Ryu et al., 2016). Assessing the importance of a declining *K*_SL_ for plant responses to soil drying requires data on characteristic parameters notably the decline rate, and the critical soil water potential at which the decline starts. Depending on these parameters a declining *K*_SL_ might contribute to varying plant responses to soil drying among species and thus account for the reduced validity of stomatal sensitivity in this context (Martínez-Vilalta et al., 2014; Martínez-Vilalta and Garcia-Forner, 2017).

Stomatal conductance and *K*_SL_, act antagonistically on the plant water status, measured as leaf water potential (Ψ_leaf_). This can be demonstrated by considering the equivalent soil water potential (Ψ_seq_) to describe the water flow from soil to leaf. Ψ_seq_ reflects the distribution of soil water potential weighted by the distribution of root conductance (Couvreur et al., 2012). Especially during droughts, when the soil water potential usually becomes heterogeneous (Hillel et al., 1976) Ψ_seq_ has a more direct relation to Ψ_leaf_ than the commonly used average soil water potential. Using Ψ_seq_, and the total RWU rate (*U*_tot_), Ψ_leaf_ can be written as

(1) $$\Psi_{\mathrm{leaf}}=\Psi_{\mathrm{seq}}-\frac{U_{\mathrm{tot}}}{\;K_{\mathrm{SL}}}.$$

During soil drying, Ψ_seq_, which depends on the soil water content, is reduced. According to Equation (1), this would lead to a drop in Ψ_leaf_. A reduction of the stomatal conductance, and thus *U*_tot_, would dampen this drop whereas a reduction of *K*_SL_ would increase it. In other words, a declining *K*_SL_ during soil drying potentially triggers stomatal closure by amplifying the drop in Ψ_leaf_. Evidence for such a coupling comes from two recent studies reporting a parallel decline of the soil–plant hydraulic conductance and the stomatal conductance during soil drying (Rodriguez-Dominguez and Brodribb, 2020; Bourbia et al., 2021). Another study proposed a strong water depletion zone around the roots to directly trigger stomatal closure (Carminati and Javaux, 2020).

Based on these findings, our study aims at answering the following questions:

1. Does soil drying cause a reduction in *K*_SL_?
2. Does the sensitivity of *K*_SL_ to soil drying vary among species?
3. Does this variation partly account for the varying stomatal sensitivity among species?
4. Does the variation in *K*_SL_ sensitivity occur in the soil or in the plant hydraulic pathway?

**Figure 1:**
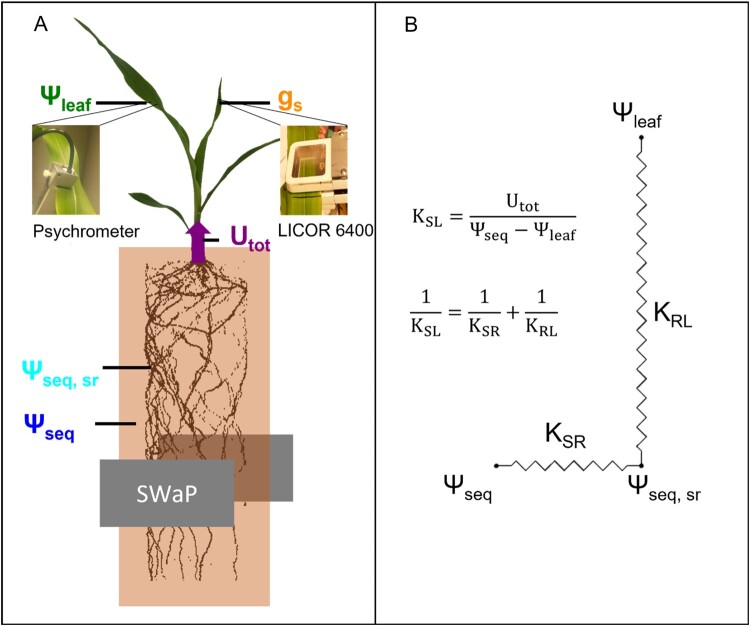
Experimental setup and hydraulic model used in this study. A, Scheme of the experimental setup and the different water potentials used for the data analysis. The equivalent water potential in the bulk soil (Ψ_seq_), at the root surface (Ψ_seq, sr_), and the total root water uptake rate (*U*_tot_) were derived from the SWaP measurements. The leaf water potential (Ψ_leaf_) was measured with psychrometers, the stomatal conductance with a LICOR 6400. Root length was determined with MRI. B, Scheme of the hydraulic network from the bulk soil to the leaf. The overall conductance from bulk soil to leaf (*K*_SL_) can be separated into the conductance from bulk soil to the root surface (*K*_SR_) and from root surface to the leaf (*K*_RL_).

We hypothesize that the differences in the stomatal response to a reduced Ψ_seq_ between two species are associated with differences in the reduction of *K*_SL_. We tested this hypothesis for two species, faba bean (*Vicia faba*), a dicot, and maize (*Zea mays*), a monocot, with different root systems and water uptake rates per unit root length. Using a highly precise soil water sensor, we continuously scanned soil water profiles during several days of progressive soil drying and derived Ψ_seq_ and *U*_tot_. In combination with Ψ_leaf_, which was measured with psychrometers, we could derive *K*_SL_ and compare it with measurements of the stomatal conductance (Figure 1). To evaluate whether the conductance between bulk soil and root surface (*K*_SR_), or the conductance inside the plant, from root surface to leaf (*K*_RL_), caused the decline in *K*_SL_, we estimated the water potential at the root surface (Ψ_sr_) using a model (van Lier et al., 2006, 2013; Carminati and Javaux, 2020).

## Results

On the first day, maize plants were significantly larger than faba bean plants regarding both leaf area (A, 1.5-fold) and root length (L, four-fold) (Table 2). Compared with maize, the smaller faba bean plants had significantly greater water uptake rates per leaf area (two-fold) and root length (six-fold). The total water uptake rate was also slightly (1.2-fold), albeit not significantly greater in faba bean. The hydraulic conductance between soil and leaf (*K*_SL_ [mL h^−1^ MPa^−1^]) was similar for faba bean and maize. Assuming that in wet soil most of the hydraulic resistance occurs in the radial pathway of the roots, the radial root conductivity can be approximated by the ratio of *K*_SL_ and *L* which was four times greater in faba bean compared with maize. As determined in a previous measurement, average root diameter was significantly greater in faba bean (0.04 cm) than in maize (0.02 cm).

**Table 2:** Characteristic plant parameters at the beginning of the water restriction period as medians ± median absolute deviation among all measured replicates

Parameter	Faba bean	Maize
*A* (m^2^)	0.036 ± 0.006^***^	0.063 ± 0.007
*L* (m)	41 ± 9^***^	162 ± 8
*U*_tot_ (mL h^−1^)	5.0 ± 0.7	4.1 ± 0.9
*K*_SL_ (mL h^−1^ MPa^−1^)	8.3 ± 1.2	7.8 ± 2.5
*U*_tot_ *A* ^−1^ (mL h^−1^ m^−2^)	138 ± 9^***^	64 ± 11
*U*_tot_ *L* ^−1^ (mL h^−1^ m^−1^)	0.119 ± 0.022^***^	0.022 ± 0.005
*K*_SL_ *L* ^−1^ (mL h^−1^ MPa^−1^ m^−1^)	0.20 ± 0.02^***^	0.05 ± 0.02

Leaf area (*A*) and root length (*L*) were determined before the start of the measurements. *U*_tot_ and *K*_SL_ were averaged across the first day of measurement (four data points per light period, excluding data from the night) for each plant. Asterisks indicate significant difference between faba bean and maize. *P*-values were derived with a Mann–Whitney *U* test. *P*-values ˂0.05 are indicated as *, ˂0.01 as ** and ˂0.005 as ***.

**Figure 2:**
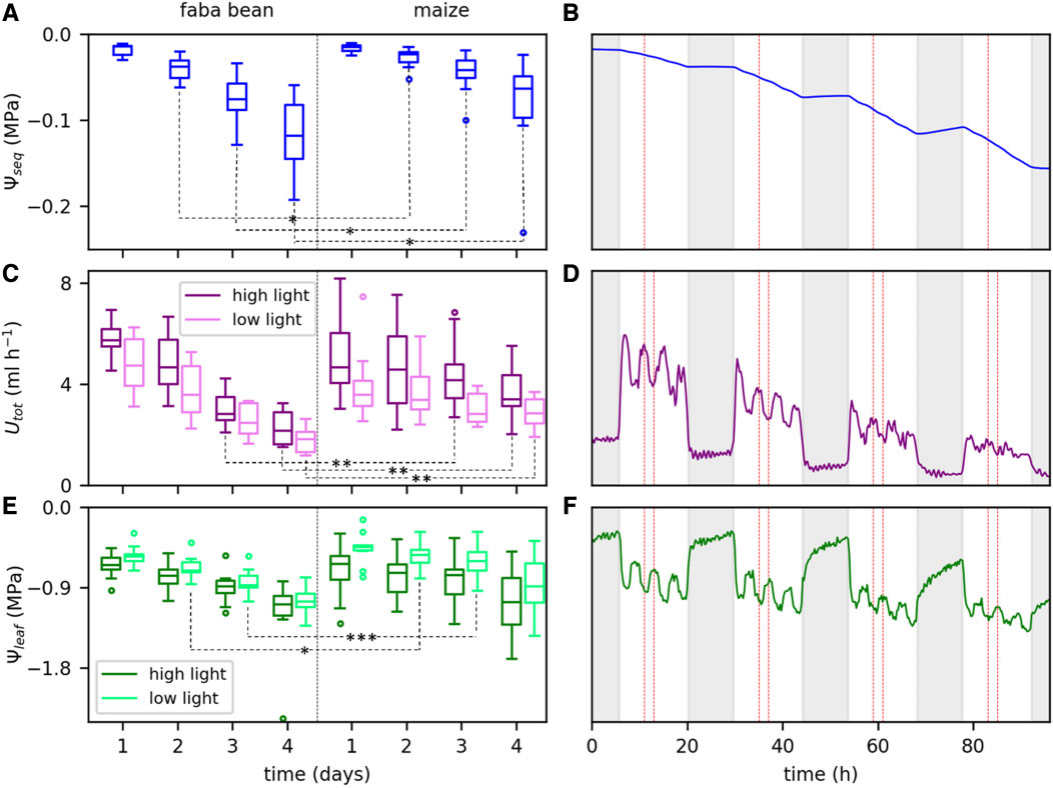
Reduction of the equivalent soil water potential (Ψ_seq_), total root water uptake rate (U_tot_), and leaf water potential (Ψ_leaf_) during 4 days of soil drying. A, C, and E, Data of all replicates (faba bean *n* = 12, maize *n* = 10) as boxplots at selected time points. Different colors in (C) and (E) refer to the two different light levels. Horizontal lines are medians, boxes reach from the first to the third quartile. Whiskers mark the minimal (lower whisker) and maximal (upper whisker) data points within 1.5 times the interquartile range from the first and third quartile, respectively. Circles are outliers beyond the whiskers. We tested for significant differences in Ψ_seq_*U*_tot_, and Ψ_leaf_ between faba bean and maize at each selected time point. *P*-values were derived with a Mann–Whitney *U* test. *P*-values ˂0.05 are indicated by *, ˂0.01 by **, and ˂0.005 by ***. B, D, and F, The continuous time courses of the three parameters during the 4 days of soil drying for one example faba bean plant. Fluctuations of the observed parameters are caused by the fluctuating light intensity. Red dashed lines mark those points used for the boxplots. Shaded areas indicate the nonilluminated periods.

Figure 2, A, C, and E, shows boxplots of Ψ_seq_, *U*_tot_, and Ψ_leaf_ at selected time points on each day during soil drying for faba bean and maize. For *U*_tot_ and Ψ_leaf_ each, one time point at low and one at high light were considered per day due to the strong light response of these two parameters. Figure 2, B, D, and F, shows the continuous time courses of the three parameters for one exemplary faba bean plant. The red vertical lines mark those time points selected for the boxplots. For faba bean, Ψ_seq_ decreased overall from −0.015 MPa on the first day to −0.12 MPa on the last day (Figure 2A). For maize, the reduction was lower, ranging from −0.015 to −0.06 MPa. The reduction of Ψ_seq_ mostly happened during the diurnal period, at a rate increasing from the first to the last day (Figure 2B). During the nights, Ψ_seq_ even increased slightly, resulting from a redistribution of soil water which Ψ_seq_ is sensitive to. *U*_tot_ decreased in faba bean from the first day on (Figure 2C) from 5.7 (high light) and 4.7 mL h^−1^ (low light) to 2.2 and 1.8 mL h^−1^ on the last day. In maize, *U*_tot_ was initially lower (4.7 mL h^−1^ at high light and 3.6 mL h^−1^ at low light) compared with faba bean and remained constant until the second day. From the third day on it also decreased to ultimately 3.4 and 2.8 mL h^−1^ and thus remained higher compared with faba bean. During the day, *U*_tot_ alternated between a higher and a lower level in response to the two different light levels. The differences in *U*_tot_ between the two light levels were lower in faba bean, especially during the last 2 days, indicating a reduced response to varying light (Figure 2D). During the night, *U*_tot_ remained constant at a low level but never zero. Also, note that *U*_tot_ at the first high light period of a day was similar to the last high light period of the previous day. This indicates that *U*_tot_ declined during the day and not at night, and thus followed the dynamics of Ψ_seq_. Ψ_leaf_ decreased gradually in faba bean (Figure 2E) from −0.7 (high light) and −0.6 MPa (low light) on the first day to −1.1 MPa and −1.0 MPa on the last day. In maize, the initial values were slightly higher (−0.6 MPa at high light and −0.5 at low light) compared with faba bean. During the following 2 days, Ψ_leaf_ only decreased slightly but more pronounced until the last day to −1.1 and −0.9 MPa. Like *U*_tot_, Ψ_leaf_ changed with the alternating light levels (Figure 2F). In contrast to *U*_tot_, however, Ψ_leaf_ did not remain constant but steadily increased during the nights. This steady increase was faster during the first compared with the last night. The first light period of a day, Ψ_leaf_ was similar (second day) or even higher (third and fourth day) compared with the last light period from the previous day.

**Figure 3:**
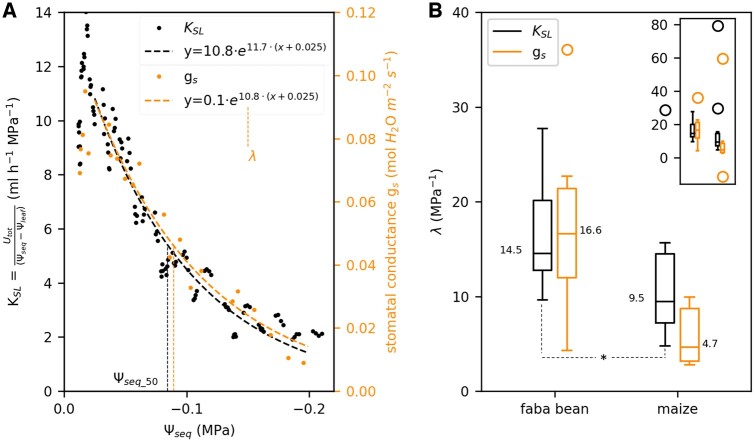
K_SL_ and *g*_s_ drop exponentially during soil drying at higher rates in faba bean than in maize. A, Example data of *K*_SL_ (black) and *g*_s_ (orange) at reducing Ψ_seq_ for one faba bean plant. Per light period we considered four measured data points of *K*_SL_ (black dots) and one measured data points of *g*_s_ (orange dots). Dashed lines follow an exponential fit of the form $K_{\mathrm{SL}}(\Psi_{\mathrm{seq}})=A\cdot e^{\lambda (\Psi_{\mathrm{seq}}+0.025)}$ or $g_{s}(\Psi_{\mathrm{seq}})=A\cdot e^{\lambda (\Psi_{\mathrm{seq}}+0.025)}$ starting at a Ψ_seq_ of −0.025 MPa. Vertical dotted lines mark the values of Ψ_seq_ at which *K*_SL_ or *g*_s_ were reduced to 50% of their initial values at a Ψ_seq_ of −0.025 MPa. B, Variation of the rate constants *λ* obtained from the exponential fits of *K*_SL_ and *g*_s_ among faba bean and maize replicates. *λ* is a measure for the sensitivity of the conductance to soil drying. Characteristics of the boxplots are similar to Figure 2, A, C, and E. Numbers on the boxes indicate the median values. The inserted figure includes all outliers which are only partly shown in the main panel. *g*_s_ was only measured for six replicates while *K*_SL_ was measured for 12 (faba bean) and 10 (maize) replicates. Asterisks indicate significant differences (*P-value < 0.05, **P-value < 0.01, ***P-value < 0.005) between faba bean and maize, tested with a Mann–Whitney *U* test.

In a next step, we analyzed the effect of soil drying on Ψ_seq_ and the hydraulic conductance between soil and leaf, *K*_SL_. *K*_SL_ was derived by rearranging Equation (1). Figure 3A shows an example *K*_SL_ (black dots) of a faba bean as a function of Ψ_seq_. For the analysis, we considered four measurement points per light period (28 points per day) excluding data measured at night. After a short, initial phase of increase, we observed an exponential decrease of *K*_SL_. Among all replicates, we found a Ψ_seq_ of −0.025 MPa as a consistent, critical point at which the exponential decline had started. For selected plants, we additionally measured the stomatal conductance *g*_s_ with a portable LiCor 6400 photosynthesis system. During the 4 days of soil drying, *g*_s_ (orange dots in Figure 3A) showed a similar dependence on Ψ_seq_ as *K*_SL_: it decreased exponentially below a Ψ_seq_ of −0.025 MPa. Note that *g*_s_ was only measured once per light period, at least four times a day. We determined the rate constant *λ* of the decline by exponentially fitting the data starting from the critical Ψ_seq_ of −0.025 MPa: $K_{\mathrm{SL}}=A\cdot e^{\lambda (\Psi_{\mathrm{seq}}+0.025)}$ or $g_{s}=A\cdot e^{\lambda (\Psi_{\mathrm{seq}}+0.025)}$. Note that Ψ_seq_ is negative and thus a positive *λ* implies a decline of *K*_SL_. Measured data on *K*_SL_ and *g*_s_ together with the exponential fit and the resulting *λ* are shown in Supplemental Figures S1 and S2 for each replicate separately. Both, *K*_SL_ and *g*_s_, declined at a higher rate in faba bean compared with maize (Figure 3B). One maize replicate (bottom left panel in Supplemental Figure S2) had a very low initial *U*_tot_ (2.5 mL h^−1^) leading to only moderate soil water depletion and thus a narrow range of Ψ_seq_ along which *K*_SL_ and *g*_s_ were fitted. This caused the strong outlier in Figure 3B. Excluding this outlier, the differences in *λ* for *K*_SL_ between faba bean and maize were significant (*P*-value < 0.05, derived with a Mann–Whitney *U* test). Among faba bean replicates, *λ* was similar for *K*_SL_ (14.5 MPa^−1^) and *g*_s_ (16.6 MPa^−1^). Note that for *K*_SL_ the median shown in Figure 3B was calculated among all 12 replicates. Stomatal conductance, however, was only measured for six of these replicates. Considering those six replicates only, the median of *λ* for *K*_SL_ is 16.0 MPa^−1^, which was not significantly different from the *λ* of *g*_s_. In maize, *λ* for *g*_s_ (4.7 MPa^−1^, Figure 3B) was lower than for *K*_SL_ (9.5 MPa^−1^). Considering only replicates for which stomatal conductance was measured results in a *λ* of 7.8 MPa^−1^ for *K*_SL_. This was significantly higher (*P*-value < 0.05) than *λ* of *g*_s_ when the outlier mentioned above was excluded.

For an alternative interpretation of the decline rates *λ*, we calculated the equivalent soil water potential at which the initial conductance was reduced to 50% (Ψ_seq_50_), as indicated by the vertical dotted lines in Figure 3A. Ψ_seq_50_ was calculated as

$$\Psi_{\mathrm{seq}\_50}=-0.025\;\mathrm{MPa}\;-\;\frac{\ln\;2}{\lambda }.$$

For faba bean, *K*_SL_ and *g*_s_ were both reduced by 50% at a Ψ_seq_ of −0.07 MPa compared with the initial value at a Ψ_seq_ of −0.025 MPa (Table 3). Maize with generally lower *λ*s, showed lower (more negative) values: Ψ_seq_50_ was −0.10 MPa for *K*_SL_ and −0.13 MPa for *g*_s_.

**Table 3:** Equivalent soil water potential at which *K*_SL_ and *g*_s_ were reduced to 50% of its initial value

Parameter	Faba bean	Maize
Ψ_seq_50_ (MPa) for *K*_SL_	−0.07 ± 0.01	−0.10 ± 0.03
Ψ_seq_50_ (MPa) for *g*_s_	−0.07 ± 0.02	−0.13 ± 0.08

Note that “initial” refers to the start of the exponential decay at a Ψ_seq_ of −0.025 MPa. Values are medians ± median absolute deviations.

To analyze how *K*_SL_ behaves on a daily scale, we determined *K*_SL_ as the slope of the relation between *U*_tot_ and Ψ_leaf_ at morning, afternoon, and evening separately (Supplemental Figure S3A). For faba bean, *K*_SL_ declined continuously during the day and was significantly lower in the evening compared with the morning on each day (Supplemental Figure S3B). However, each morning, *K*_SL_ tended to be greater than on the previous evening, consistent with the increasing Ψ_seq_ during the nights (Figure 2B). For maize, we also observed consistently lower *K*_SL_ in the evening compared with the morning of the same day, but on some days *K*_SL_ slightly increased from morning to afternoon or from afternoon to evening.

**Figure 4:**
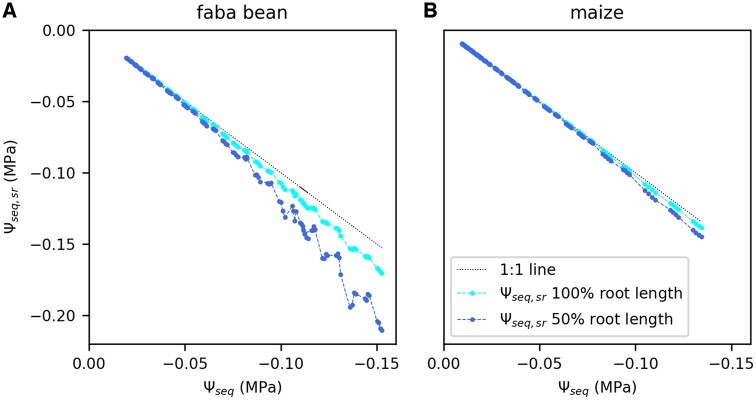
Estimated water potential at the root surface (Ψ_seq, sr_) as a function of the bulk soil water potential (Ψ_seq_). Data are an example shown for one faba bean (A) and one maize plant (B). Water potential at the root surface was calculated for the full measured root length and half of the measured root length. Dotted line is the 1:1 line.

The hydraulic pathway from bulk soil to the leaf can be separated into a soil part (from bulk soil to the root surface) and a plant part (from root surface to the leaf). Here, we want to estimate whether the observed reduction in *K*_SL_ mostly happened in the soil or in the plant pathway. Deriving the hydraulic conductance of each part separately requires the water potential at the root surface Ψ_seq_, _sr_ to be known. Ψ_seq_, _sr_ can differ from Ψ_seq_ (referring to the bulk soil) due to a water depletion zone around the roots which can be estimated using a model (Carminati and Javaux, 2020). We estimated Ψ_seq_, _sr_ for two different scenarios: (1) the full root length is actively involved in water uptake. (2) Only 50% of the root length takes up water. Figure 4 shows an example calculated Ψ_seq, sr_ in comparison to Ψ_seq_ for one faba bean (Figure 4A) and one maize plant (Figure 4B). Generally, the difference between Ψ_seq, sr_ and Ψ_seq_ increases with increasing water uptake rates per unit root length and decreasing Ψ_seq_. For faba bean, considering 100% root length (cyan), there was only a marginal difference between Ψ_seq, sr_ and Ψ_seq_ (black 1:1 line) (Figure 4A). Conservatively assuming that only 50% of the root length is active in water uptake led to a generally lower Ψ_seq, sr_ (blue). In this scenario, Ψ_seq, sr_ showed some fluctuations at the end of the measurement (Figure 4A) due to the dependency on the transpiration rate and thus the alternating light intensity. The difference between Ψ_seq, sr_ and Ψ_seq_ was still low (˂0.02 MPa) until a Ψ_seq_ of −0.10 MPa. At the end of the measurement, Ψ_seq, sr_ was 0.05 MPa lower than Ψ_seq_. For maize, in both scenarios, the differences between Ψ_seq, sr_ and Ψ_seq_ were negligible.

**Figure 5:**
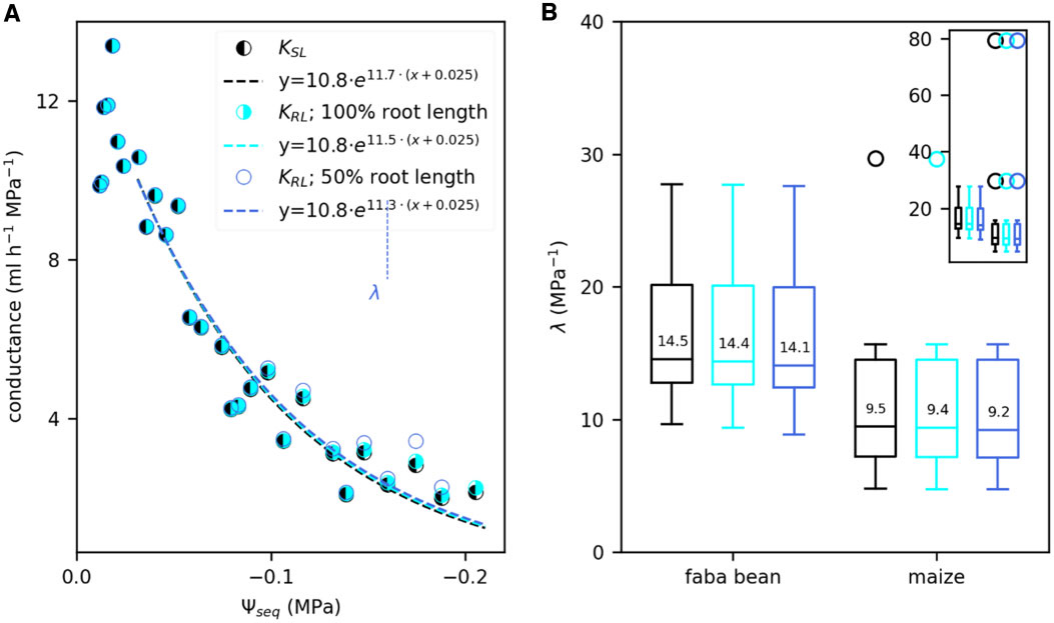
Comparison between the hydraulic conductance between root surface and leaf (*K*_RL_) and *K*_SL_ throughout the soil drying period. A, *K*_RL_ compared with *K*_SL_, as a function of Ψ_seq_, example shown for one faba bean plant (same plant as in Figure 3A). *K*_RL_ was determined using the calculated water potential at the root surface for the full root length (cyan) or half of the root length (blue). We exponentially fitted the data (dashed lines) to determine the rate constants *λ* starting at a Ψ_seq_ of −0.025 MPa. For reasons of clarity, we plotted only one data point per light period here but derived the exponential fit using four data points per light period, analogously to Figure 3. B, Boxplots of the rate constants *λ* obtained from the exponential fits of *K*_SL_ (black, same data as in Figure 3B) and *K*_RL_ considering the full root length (cyan) or half of the full root length (blue) for faba bean (*n* = 12) and maize (*n* = 10). Characteristics of the boxplots are similar to Figure 2, A, C, and E. The main panel does not include all outliers which are shown in the inserted figure.

Using the calculated Ψ_seq, sr_, we could derive the conductance between bulk soil and root surface and between root surface and leaf (*K*_RL_) separately. For this, we divided the total water uptake rate by the water potential difference between bulk soil and root surface and root surface and leaf, respectively. Since the estimation of Ψ_seq, sr_ does not account for a loss of soil–root contact, the conductance of the interface between soil and root is included in *K*_RL_. In the following, we compare *K*_RL_ to the overall conductance *K*_SL_. For one faba bean replicate, Figure 5A shows an example *K*_SL_ (black, half-filled circles) and *K*_RL_ for 100% root length (cyan, half-filled circles) and 50% root length (blue, nonfilled circles). Over a broad range of Ψ_seq_, *K*_SL_ and *K*_RL_ were almost identical, both showing the above-described exponential decay. Only below a Ψ_seq_ of −0.18 MPa, *K*_RL_ (50% root length) remained considerably higher than *K*_SL_ and even increased slightly. Note that at this point, *K*_SL_ was already reduced by 75%. Analogously to *K*_SL_ we quantified the decay of *K*_RL_ by determining the decay rate *λ* of an exponential fit. *λ* of *K*_RL_ was close to that of *K*_SL_ in all crops and simulated scenarios (Figure 5B).

These results suggest that the decline in *K*_SL_ is almost completely explained by a decline in *K*_RL_ and the effect of a reduced Ψ_seq, sr_ was negligible. For further evidence, we created the hypothetical, opposite scenario and tested how strong Ψ_seq, sr_ would need to drop to fully explain the measured decline in *K*_SL_ (Supplemental Figure S4). The theoretical Ψ_seq, sr_ was calculated using a variation of Equation (1) for the pathway between root surface and leaf:

(2) $$\Psi_{\mathrm{seq},\;\mathrm{sr}}=\Psi_{\mathrm{leaf}}-\frac{U_{\mathrm{tot}}}{\;K_{\mathrm{RL}}}.$$

For this scenario, *K*_RL_ was assumed to be constant and approximated by the initial *K*_SL_ at Ψ_seq_ = −0.025 MPa. The calculation shows that if *K*_RL_ was constant, Ψ_seq, sr_ would need to decrease ˂−0.6 MPa at a Ψ_seq_ of −0.1 MPa and ˂−1.2 MPa at a Ψ_seq_ of −0.2 MPa to account for the reduction in *K*_SL_. Then, we tested how close the estimated Ψ_seq, sr_, using the water depletion model, could get to this hypothetical line by considering only a reduced fraction of root length or root diameter (Supplemental Figure S4). For none of the tested fractions, the estimated Ψ_seq, sr_ was comparable to the theoretical one at constant *K*_RL_ either in terms of amplitude or shape of the decline. This analysis supports our conclusion that most of the decline in *K*_SL_ did not occur between bulk soil and root surface.

We compared the root architectures between faba bean and maize to further elucidate the different responses to soil drying between the species. As an example shown in Supplemental Figure S5A, faba bean had a greater fraction of root length in the top 10 cm while in maize the fraction ˂30 cm was greater. For quantification, we determined the depth *D*_50_, at which 50% of the total root length was reached, which was significantly deeper in maize (20 cm, Supplemental Figure S5D) than in faba bean (12 cm). The resulting pattern of water uptake rates (${\hat{U}}_{P}$) was initially contrasting, with a greater fraction of water uptake in shallow layers for maize compared with faba bean (Supplemental Figure S5B). This changed toward the last day of measurement when the fraction of ${\hat{U}}_{P}$ ˂30 cm was greater in maize than in faba bean (Supplemental Figure S5C). These observations were confirmed by the D_50 of ${\hat{U}}_{P}$, which was deeper in faba bean for the first day but shifted to a significantly deeper layer in maize for the last day (Supplemental Figure S5D).

**Figure 6:**
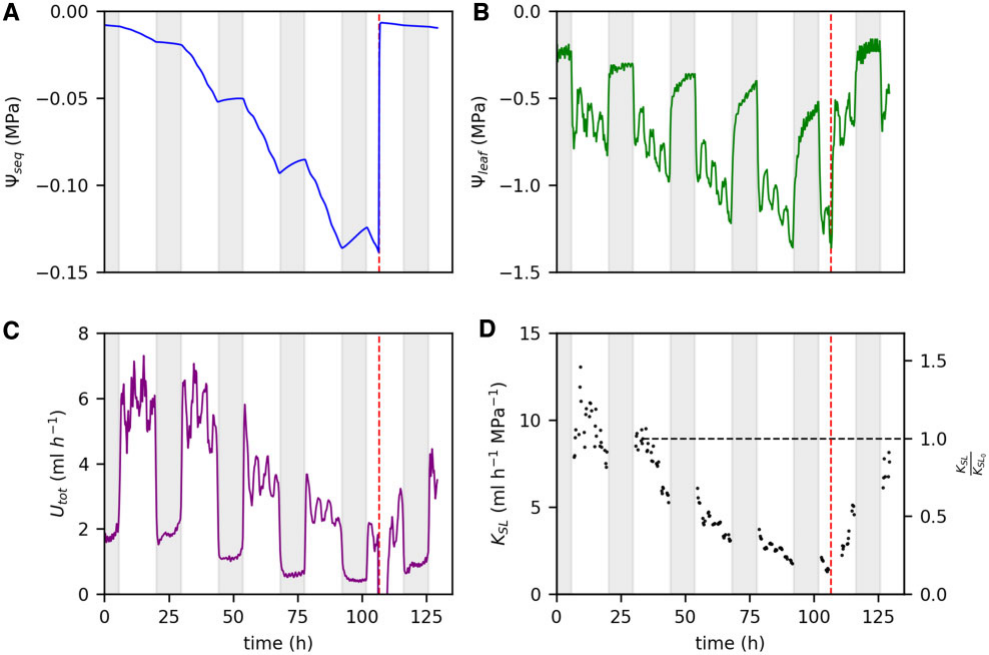
Recovery of different hydraulic parameters upon rewatering after 4 days of water restriction for one faba bean plant. Parameters are Ψ_seq_ (A), Ψ_leaf_ (B), *U*_tot_ (C), and *K*_SL_ (D). Vertical dashed lines mark the time point of rewatering. Horizontal dashed line in (D) marks the initial value of *K*_SL_ at a Ψ_seq_ of −0.025 MPa (*K*_SL,0_). Nights are indicated by the shaded areas.

After 4 days of soil drying, we rewatered four of the faba bean plants to analyze how the measured parameters would recover. The example in Figure 6 shows the data for one faba bean plant. Within 30 min after rewatering, Ψ_seq_ increased from −0.14 to −0.01 MPa (Figure 6A) which is comparable to the initial value on the first day. Within 1 h after rewatering, Ψ_leaf_ increased from −1.3 to −0.8 MPa (Figure 6B) which was only slightly lower compared with the initial Ψ_leaf_ of −0.7 MPa. During the next 20 h, Ψ_leaf_ further increased resulting in values of −0.6 MPa which is even lower compared with the start of the measurement. In contrast, the recovery of *U*_tot_ was slower (Figure 6C): 5 h after rewatering, *U*_tot_ at high light level was similar to the high light level before rewatering (around 1.9 mL h^−1^). Four hours later, *U*_tot_ had increased to 3.1 mL h^−1^. During the following night, *U*_tot_ further increased steadily and reached around 4.0 mL h^−1^ on the next morning (21 h after rewatering) which is around 40% lower compared with the initial values on the first day (6.5 mL h^−1^). Compared with the value at a Ψ_seq_ of −0.025 (horizontal dotted line in Figure 6D), *K*_SL_ had declined to around 17% before rewatering (Figure 6D). Upon rewatering, *K*_SL_ steadily increased, up to 50% of the initial value after 8 h. The next morning, within 20 h after rewatering, *K*_SL_ had recovered to around 85%. For the other three rewatered faba bean plants, measurements were taken only up to 5.5 h after rewatering. Nevertheless, trends were similar (Supplemental Figure S6, A–F): Ψ_leaf_ reached the initial value within several hours after rewatering while *U*_tot_ recovered much slower. Data from a separate experiment on two 6-weeks-old faba bean plants indicate that full recovery of *U*_tot_ took 40 h after rewatering (Supplemental Figure S6, G–H).

## Discussion

The hydraulic conductance between soil and leaf declined exponentially with reducing soil water potential. In faba bean, this decline occurred at a higher rate than in maize which partly explains that faba bean closed its stomata more quickly. Estimations of the water potential at the root surface suggest that not only the soil conductance but also the conductance between root surface and leaf dropped. This was supported by an incomplete recovery, 22 h after rewatering.

We analyzed to what extent the hydraulic conductance between soil and leaf (*K*_SL_) is affected by soil drying. For that we continuously and noninvasively measured the total root water uptake rate (*U*_tot_) and the equivalent water potential in the bulk soil (Ψ_seq_) with the soil water profiler (SWaP) and the leaf water potential (Ψ_leaf_) on faba bean and maize during 4 days of soil drying. The initial conductance at the beginning of soil drying was higher in faba bean (8.3 mL h^−1^ MPa^−1^, or 3.5 mmol m^−2^ s^−1^ MPa^−1^ when normalized by leaf area) than in maize (7.8 mL h^−1^ MPa^−1^ or 1.9 mmol m^−2^ s^−1^ MPa^−1^). These values fit in the range reported in the literature for lupine (*Lupinus albus* L.) (13.7 mL h^−1^ MPa^−1^; Hayat et al., 2019) *Tanacetum cinerariifolium* and *Callitris rhomboidea* (both around 4.5 mmol m^−2^ s^−1^ MPa^−1^; Bourbia et al., 2021) or olive (*Olea europaea* L. var. *arbequina*) (0.7 mmol m^−2^ s^−1^ MPa^−1^; Rodriguez-Dominguez and Brodribb, 2020). Among replicates, *K*_SL_ consistently declined exponentially as a function of the equivalent soil water potential starting at a Ψ_seq_ of −0.025 MPa (Figure 3A). We determined the rate constant *λ* of this decline by exponentially fitting *K*_SL_ (Ψ_seq_). The rate constant is a measure for how sensitive the conductance is to soil drying: A high *λ* reflects a fast decline of the conductance and thus high sensitivity to a reducing Ψ_seq_. Among faba bean replicates we found a *λ* of 15 MPa^−1^. This is equivalent to 50% reduction of *K*_SL_ within a range of Ψ_seq_ from −0.025 to −0.07 MPa. In maize, *λ* was lower (10 MPa^−1^) and *K*_SL_ dropped to 50% only at a Ψ_seq_ of −0.10 MPa. These high decline rates are probably caused by the sandy soil substrate. Hayat et al. (2019) reported similar values for lupine plants in a sandy soil (90% loss of conductance at a Ψ_seq_ of −0.17 MPa). A study among different sugarcane (*Saccharum* spp. hybrid) cultivars reports an even faster decline, with an almost complete loss of conductance before Ψ_soil_ reached −0.1 MPa (Saliendra and Meinzer, 1989). On the other hand, slower declines have been observed as well, ranging from a 50% loss of conductance below a Ψ_soil_ of −1.0 MPa in *T. cinerariifolium* and *Callitris rhomboidei* (Bourbia et al., 2021) to a 30%–60% loss only below a Ψ_soil_ of −10 MPa in desert succulents (Nobel and Cui, 1992). The rate constants observed in our study highlight the impact of the *K*_SL_ decline on the plant response to soil drying: For faba bean, sustaining the initial transpiration rate, and thus stomatal opening, would lead to a drop in Ψ_leaf_ as little as −0.045 MPa to compensate the reduced Ψ_seq_ from −0.025 to −0.07 MPa. Due to the 50% reduction of *K*_SL_, however, keeping the stomata open would result in a much greater drop in Ψ_leaf_ by −0.6 MPa (see Equation (1)). Given these values, it is highly likely that the drop of *K*_SL_ affects stomatal conductance. Note that for the considerations described above, we referred to the water potential in the bulk soil. Whether a drop of the water potential around the roots accounted for the observed drop in *K*_SL_ is discussed further below.

The stomatal conductance (*g*_s_), measured with a portable LiCor 6400 photosynthesis system for selected plants, also declined exponentially starting at a Ψ_seq_ of −0.025 MPa (Figure 3A). Again, we quantified the exponential decay by determining the rate constant *λ*. In the case of *g*_s_, *λ* is an estimate for how strong the stomata respond to soil drying. A higher *λ* indicates a higher reduction of the stomatal conductance at a given Ψ_seq_, independent of how fast Ψ_seq_ was reduced. Like for *K*_SL_, the decline rate for *g*_s_ was lower in maize compared with faba bean. This can be interpreted as follows: at a given level of soil drying, measured as Ψ_seq_, faba bean experienced a stronger reduction of water availability than maize due to the stronger decline of the hydraulic conductance between bulk soil and leaf. This led to a stronger reduction of the stomatal conductance. The exact mechanism which couples *K*_SL_ and *g*_s_ is still unclear. Different, nonhydraulic signaling cascades such as enhanced abscisic acid biosynthesis (Liang et al., 1997), reduced cytokinin supply (Blackman and Davies, 1985), or suppressed stringolactone biosynthesis (Visentin et al., 2016) have been suggested to propagate from dehydrated roots to the shoot and initiate stomatal closure. In this case, *K*_SL_ and *g*_s_ would be linked. A recent review, however, concludes that most of the stomatal regulation happens hydraulically via the leaf water potential (Buckley, 2019). We, therefore, postulate that *K*_SL_ and *g*_s_ are indirectly linked by the balancing of Ψ_leaf_ and the transpiration rate (Equation (1)) although our data do not allow a clear distinction between transpiration rate and *g*_s_. Both were closely linked since the VPD was kept constant by the climate chamber control and water-cooling of the LED panel. Irrespective of the above, our data suggest that the variation of stomatal sensitivity among species can partly be attributed to a variation of the *K*_SL_ sensitivity. Potential causes for the variation of the *K*_SL_ sensitivity among species are discussed below. However, while in faba bean, *K*_SL_ and *g*_s_, in agreement with several recent studies (Rodriguez-Dominguez and Brodribb, 2020; Abdalla et al., 2021; Bourbia et al., 2021), declined almost in parallel, in maize *g*_s_ declined at a 50% lower rate than *K*_SL_ (Figure 3B). This indicates that in addition to its dependence on *K*_SL_, stomatal sensitivity is partly a species-inherent trait as commonly assumed (Tardieu and Simmoneau, 1998; Klein, 2014).

We used Ψ_seq_ as a measure for the extent of soil drying that the plant is exposed to. Ψ_seq_ is the distribution of soil water potential weighted by the distribution of root conductance (${\hat{U}}_{P}$) which is directly linked to the root distribution. During the 4 days of soil drying, Ψ_seq_ was generally lower in faba bean than in maize, especially on the last 2 days (Figure 1A). Since the total water uptake rates were comparable or, during the last 2 days even greater in maize (Figure 1C), the differences in Ψ_seq_ are most likely explained by the different root architectures between the two species: maize had a higher fraction of deep roots than faba bean (Supplemental Figure S5, A and D) and thus was able to acquire a higher fraction of water from deeper layers at the later stages of the experiment (Supplemental Figure S5, C and D). Since the soil water potential usually is less negative in those deeper layers, the weighted Ψ_seq_ was less negative in maize than in faba bean. Note that by using Ψ_seq_, we account for the effect of root distribution on *K*_SL_ which is not case when the more common average soil water potential or soil water content are used as a measure for soil drying.

Explaining the different rate constants between faba bean and maize starts with determining which part of the hydraulic pathway between bulk soil and leaf caused the observed drop in *K*_SL_. Recently, a water depletion zone around the roots has been proposed to account for a major loss of hydraulic conductance from soil to plant which could initiate stomatal closure (Carminati and Javaux, 2020). The water depletion zone would cause a much lower water potential at the root surface Ψ_seq, sr_ than in the bulk soil Ψ_seq_. Based on a model by Carminati and Javaux (2020), Ψ_seq, sr_ can be calculated for given soil hydraulic properties. The difference between Ψ_seq, sr_ and Ψ_seq_ increases with increasing water uptake rate per root length, decreasing root radius, and decreasing Ψ_seq_. We observed a steeper decline of Ψ_seq, sr_ with Ψ_seq_ in faba bean than in maize. Since the total water uptake rates were comparable between the two species, the differences in Ψ_seq, sr_ are caused by differences in the total root length and the average root diameter. Faba bean had a greater average root radius but a smaller total root length than maize (Table 2) which is typical since dicots usually have a lower specific root length than monocots (Read et al., 2010). As demonstrated in Supplemental Figure S4, root length had a stronger impact on the estimated Ψ_seq, sr_ than diameter. Therefore, the steeper decline of Ψ_seq, sr_ in faba bean is explained by the much lower total root length compared with maize. Nevertheless, the calculated difference between Ψ_seq, sr_ and Ψ_seq_ (Figure 4) and thus the impact on the decline of *K*_SL_ was marginal for both faba bean and maize (Figure 5). In another study, the drop of Ψ_seq, sr_ could explain experimental data on the loss of soil–plant conductance when only 0.7%–2.5% of the measured root length was considered in the water uptake process (Hayat et al., 2020). From the magnetic resonance imaging (MRI) images, we know that parts of the root system were close to the pot borders in our experiment. This might have limited the access to soil water leading to a reduction of the active root length, however, not to such a drastic extent. Nevertheless, we tested the effect of halving the measured root length which resulted in a considerably lower Ψ_seq, sr_ for faba bean at the end of the measurements (Figure 4) but had no remarkable impact on *K*_SL_ (Figure 5). This indicates that even though the soil conductivity around the roots drops sharply (Supplemental Figure S7) at the measured soil water regimes, it stays considerably greater than *K*_RL_. Therefore, our data suggest that most of the *K*_SL_ decline did not occur in the soil zone close to the roots. Note that we used a total root length and average root radius for the estimation of Ψ_seq, sr_ and did not consider the spatial root distributions. Nevertheless, our analysis shows that even though we probably overestimate local root length densities or root radius, the model estimations of Ψ_seq, sr_ are far from explaining the decline in *K*_SL_ (Supplemental Figure S4).

To further clarify whether the drop of *K*_SL_ occurred inside or outside the plant, we need to consider a potential loss of the soil–root contact. Dehydration of root tissue in drying soils can lead to root shrinkage and thus enhance the reduction of the soil–root contact (Nobel and Cui, 1992; North and Nobel, 1997; Carminati et al., 2009). The estimation of Ψ_seq, sr_ does not account for a reduced root soil contact. Therefore, the estimated decline of *K*_RL_ (Figure 5) includes the decreasing conductance resulting from a potential shrinkage of roots. Rodriguez-Dominguez and Brodribb (2020) observed a reduced conductance of the soil–root interface, attributed to root shrinkage, to mainly cause a 74% drop in the overall plant conductance during soil drying. Compared with our study, this drop is in the same order of magnitude but occurred at a much lower water potential (Ψ_stem_ between −1.0 and −4.0 MPa (Rodriguez-Dominguez and Brodribb, 2020) compared with Ψ_leaf_ between −0.6 and −1.1 MPa in our study (Figure 2)). However, X-ray CT studies on lupin (Carminati et al., 2013), faba bean (Koebernick et al., 2018), and maize (Duddek et al., 2022) revealed substantial root shrinkage leading to air gaps between soil and root starting already at a relatively high Ψ_soil_ of −0.01 to −0.02 MPa. This is comparable to the critical Ψ_seq_ of −0.025 MPa at which the decline of *K*_SL_ started in our study. Carminati et al. (2013) suggested that a slightly reduced soil conductivity led to the initial dehydration and shrinkage of the roots, which then, in a self-enhancing process, would cause an additional drop of the conductance and thus additional root shrinkage. In turn, the temporary recovery of *K*_SL_ that we observed during the night (Supplemental Figure S3) could result from root rehydration and thus a recovery of soil–root contact. This would fit the observation of a diurnal variation of root diameter with shrinkage during the day, and swelling during the night (Huck et al., 1970). However, in faba bean, root shrinkage was shown to be almost fully reversed within 3 h after rewatering (Koebernick et al., 2018). In our study, the *K*_SL_ of faba bean had not fully recovered within 20 h after rewatering (Figure 6D and Supplemental Figure S6) while Ψ_seq_ was almost fully recovered within 30 min (Figure 6A). This suggests that a reduction of the conductance between bulk soil and root surface was not the only reason for the reduced *K*_SL_.

It is, therefore, likely that the drop of *K*_SL_ partly occurred inside the plant including the radial pathway from root surface into the root xylem and the axial pathway from root xylem into the leaf. Root aquaporin activity has been shown to modulate the loss of root hydraulic conductance during soil drying for various species (Martre et al., 2001; Aroca et al., 2006; Galmés et al., 2007; Perrone et al., 2012; Grondin et al., 2016; Rodríguez-Gamir et al., 2019). Other studies report a close linkage between a reduced radial root conductivity and suberization of the root endodermis (Cruz et al., 1992; Lo Gullo et al., 1998) or lacunae formation in the tissue of fine roots (Cuneo et al., 2016) or both (North and Nobel, 1991). Whereas the aquaporin contribution to root conductance was shown to fully recover within 5 h after rewatering (Rodríguez-Gamir et al., 2019), lacunae formation (Cuneo et al., 2016) and suberization (Lo Gullo et al., 1998) are permanent and require growth of new roots to restore *K*_SL_. This could explain the incomplete recovery of *K*_SL_ 20 h after rewatering (Figure 6D) in our study. The extent of reduced conductance caused by changes in gene expression and root morphology varies among species. This is highlighted by two grapevine cultivars, for which differences in the reduction of root hydraulic conductance could be assigned to a difference in aquaporin expression during drought (Vandeleur et al., 2009). Another study on two different grapevine rootstocks revealed a stronger decline of root hydraulic conductance to correspond to a faster suberization (Barrios-Masias et al., 2015). If such variations occur even within the same species, it is likely that the different rate constants of *K*_SL_ between faba bean and maize in our study are partly caused by differences in root morphological changes upon soil drying.

Although xylem embolism is predominantly observed in trees below a stem water potential of −2.0 MPa (Cochard, 2006), some studies suggest that it also needs to be considered in crops: in maize, xylem embolism was shown to cause a 25% loss of conductance in leaves at a Ψ_soil_ of −0.25 MPa (Ryu et al., 2016) and 23% loss of conductance in stems at a Ψ_stem_ of −1.0 MPa (Li et al., 2009). Another study on maize leaves, however, shows that the conductance loss due to xylem embolism is less than 15% until a Ψ_stem_ of −1.5 MPa is reached (Cochard, 2002). Since in our experiments, Ψ_leaf_ mostly remained >−1.2 MPa (Figure 2E) and the decline of *K*_SL_ started at a Ψ_seq_ of −0.025 MPa, we conclude that a reduced xylem conductance due to embolism did not cause the decline in *K*_SL_.

In summary, our estimations of Ψ_seq, sr_ and the slow recovery after rewatering indicate that the decrease in *K*_SL_ partly occurred in the hydraulic pathway between root surface and leaf, and thus inside the plant. This should not obscure the fact that a reduction in the soil water potential around the roots with, however only a marginal effect on *K*_SL_, is likely to be the initial cause leading to a decreasing plant hydraulic conductance. The greater extent of water depletion around faba bean roots compared with maize roots at a given Ψ_seq_ (Figure 4) could have led to a stronger response of *K*_SL_ in faba bean. From this perspective, the significant differences in root length densities (Table 2) might be the crucial factor to explain the differences of the decline in *K*_SL_, and ultimately in *g*_s_, between the two species. Additionally, faba bean generally had a greater initial stomatal conductance than maize (Supplemental Figures S1 and S2) which also contributed to the initially greater water uptake rates per unit root length (Table 2). The resulting dehydration of the root tissue could then have triggered a decrease of radial root conductance by alterations in the gene expression (aquaporins) or in root morphology (suberization) which amplifies the reduction of *K*_SL_. But why would the plant initiate such a drastic decline of *K*_SL_ when the water supply toward the roots is only moderately constrained? As suggested by Carminati et al. (2020), stomatal closure at an early stage of water stress can prevent a severe drop of the water potential around the roots. Since leaf water status seems to be the principal factor regulating stomatal conductance (Buckley, 2019), we speculate that the decrease in *K*_SL_ is a mechanism to force stomatal closure by enhancing the drop in Ψ_leaf_. Such a strategy would amplify the initial water stress but could be beneficial in the long-term by avoiding a steep water potential gradient toward the root surface. In addition, the decrease of *K*_SL_ in roots in drier soil layers could be accompanied by root growth in wetter soil layers. This would lead to lower water uptake rates per unit root length and thus help to avoid excessive rhizosphere drying. However, it should be kept in mind that due to the potential loss of soil–root contact, it remains difficult to evaluate to what extent the plant is in control of the *K*_SL_ decline. Despite this speculative aspect, our study clearly highlights that how a plant responds to water stress, strongly depends on how sensitive *K*_SL_ is to soil drying. This has different implications on the widely used Feddes model (Feddes et al., 1978; Feddes et al., 2001), at least when plants grow in a loamy sand:

1. The range of Ψ_seq_ at which the transpiration rate is sustained is very narrow because *K*_SL_ starts already declining at Ψ_seq_ = −0.025 MPa.
2. The slope of the declining part of the water stress curve is not expected to be constant anymore since it depends on *K*_SL_ (Couvreur et al., 2015).
3. The point at which transpiration reaches zero is much higher (less negative) than the permanent wilting point of −1.5 MPa.
4. The extent of reduced water availability at a given Ψ_seq_ is not constant anymore but varies among species due to a varying *K*_SL_ sensitivity to soil drying.

To obtain a more precise description of plant responses to soil drying, the reduction of *K*_SL_ should be taken into account when calculating water stress curves. This could be realized by the rate constant *λ* of the declining *K*_SL_(Ψ_seq_) which, however, varies among species.

## Conclusion

We observed a strong reduction of the hydraulic conductance between soil and leaf, *K*_SL_, at even moderately low soil water potentials. This implies that the main hydraulic impairment during soil drying does not only result from the reduced soil water potential but from the reduced hydraulic conductance between soil and leaves. Therefore, how plants respond to a gradually reduced soil water potential strongly depends on the extent of the *K*_SL_ reduction. Here, we show that between two species, faba bean and maize the sensitivity of *K*_SL_ differs as quantified by the rate constant of the exponential decline. In faba bean, this rate constant is higher than in maize implying a faster reduction of the water availability at a given soil water potential and thus a stronger impulse for stomatal closure. In agreement with that, also the stomatal conductance declined at a higher rate in faba bean than in maize suggesting that varying stomatal sensitivity among species partly arises from a varying susceptibility of *K*_SL_. A potential origin for the differences in the *K*_SL_ susceptibility could be the initial water uptake rate per root length which was six times higher in faba bean than in maize. Our data suggest that the *K*_SL_ decline partly occurs inside the plant which could be a strategy to avoid a severe water depletion zone around the roots and thus improve plant performance during a longer drought. However, additional studies are needed to further disentangle the role of the soil and plant in response to drought. This could be realized by comparing the *K*_SL_ decline among plants grown in different soils with varying hydraulic properties.

## Materials and methods

### Plant growing conditions and experimental design

Faba bean (*V. faba*, *n* = 12) and maize (*Z. mays*, *n* = 10) plants were grown in PVC pipes (50 cm high, inner diameter: 8.1 cm) filled with a sandy loam containing 73.3% sand, 23.1% silt, 3.6% clay, as reported by Pohlmeier et al. (2009), mixed with 20% (v) coarse sand (0.7–1.4 mm). The water retention curve of the substrate is shown in Supplemental Figure S7. A total substrate volume of 2.32 L was filled into the pots to a height of 45 cm resulting in a bulk density of 1.47 kg/L. Plants were grown in a climate chamber at the Research Centre Jülich under a constant temperature of 21.5°C ± 0.2°C and a VPD_air_ of 1.49 kPa. Until the start of the measurements, plants were regularly watered to maintain an average volumetric soil water content (*θ*) of around 20%. Once a week, plants were fertilized using an NPK nutrient salt (Hakaphos Red; Compo Expert; 8% N, 12% P, 24% K), diluted in water at 0.3% (v/v). Plants were illuminated using a water-cooled LED panel (3200K, 5 × 5 LEDs 20W each) for 14 h during the day. Within these 14 h, light intensity was regulated to alternate in 2-h periods of a high (PPFD of 1,000 µmol m^−2^ s^−1^) and a low light intensity (PPFD of 500 µmol m^−2^ s^−1^) resulting in a daily light integral of 39.6 mol m^−2^ d^−1^. The alternating light pattern enabled us to derive water uptake profiles with the SWaP as described below. At an age between 4 and 5 weeks after sowing, selected according to a preferably similar water uptake rate among replicates, root system of the plants were imaged with MRI. Then, water supply was withheld for 4 days. During these 4 days *U*_tot_ and Ψ_seq_ were determined with the SWaP. Simultaneously, Ψ_leaf_ and the leaf gas exchange were measured with a psychrometer and a LiCor 6400, respectively. After the 4 days of soil drying, some of the plants were rewatered to observe if *U*_tot_, Ψ_seq_, and Ψ_leaf_ recovered.

### Root length measurement with MRI

Right before the start of the soil drying experiment, root length distributions of the plants were determined using noninvasive imaging with MRI. A 4.7T vertical wide bore (310 mm) magnet (Magnex, Oxford, UK) and a gradient coil (ID 205 mm [MR Solutions]) generating gradients up to 400 mT/m were used in our setup. An MR Solutions (Guildford, UK) console was used to control the measurements. MRI data were analyzed with NMRooting software (van Dusschoten et al., 2016) yielding the total root length and root length distributions at a 1-cm vertical resolution. For a similar MRI setup, roots with a diameter ˂200–300 µm were below the detection limit (van Dusschoten et al., 2016). In an earlier experiment with 4-weeks-old faba bean and maize plants, grown under the same conditions, we compared a destructive measurement (harvest and scanning roots) of the total root length to the noninvasive measurement with MRI and NMRooting. For faba bean, 70.5% of the destructively determined root length was detected with MRI. For maize with generally thinner roots, 18.0% were detected. To correct for that, we multiplied the total root length measured with MRI in this study by the respective correction factors (1.4 for faba bean and 5.6 for maize). Additionally, we obtained the average root radius (*r*_0_) of both species from the scanning of harvested roots in the earlier experiment.

### SWaP measurements of *U*_tot_ and Ψ_seq_

We used the recently developed SWaP (van Dusschoten et al., 2020) to continuously scan the profile of the volumetric soil water content (*θ*) which enabled us to derive both *U*_tot_ and Ψ_seq_. In principle, the SWaP measurement is based on integrating the pots with the soil columns into a resonator circuit and then determining the resonance frequency which largely depends on *θ*. To this end, a sensor with two opposing copper plates (7 × 5 cm^2^) coupled to a coil partially encloses the pots with soil. The resonance frequency is determined by applying a frequency sweep between 150 and 220 MHz using a virtual network analyzer (DG8SAQ, VNWA3, SDR-Kits, UK). The sensor moves upward the pots in 1-cm steps and determines the resonance frequency at each step. This yields a vertical profile of the resonance frequency consisting of 45 values. The profiles were measured every 15 min which, for four pots simultaneously, took around 11 min. The sensors were calibrated using soil samples with a defined *θ* ranging from 2% to 30% in 2% steps which enabled us to transfer the resonance frequency profiles into *θ* profiles. Since the sensors have a height of 12 cm and measurements were taken in 1-cm steps, the measured *θ* value in each layer is a convolution of the sensors’ field strength distribution and the *θ* values from the adjacent layers. We, therefore, applied a deconvolution of the measured *θ* profiles. To avoid an error amplification by the deconvolution, we used a regularization term to constrain the deconvolved profiles.

For the following analysis, we treat the 45-cm high soil column as consisting of 45 stacked soil layers of 1 cm height, each with a uniform *θ*. Since both evaporation from the topsoil, which was covered with plastic, and water drainage at the bottom of the pots were negligible we could derive *U*_tot_ as the sum of water depletion rates in each layer:

(3) $$U_{\mathrm{tot}}\left(t\right)=\sum_{i=1}^{45}\frac{\partial \theta \left(z_{i},t\right)}{\partial t},$$

with time *t* and depth of each layer *z_i_*, ranging from 0 to 44 cm.

The equivalent soil water potential (Ψ_seq_) as proposed by Couvreur et al. (2012) is the distribution of soil water potential (Ψ_soil_) weighted by the plant-driven RWU distribution (${\hat{U}}_{P}$) which is the distribution of root hydraulic conductance:

(4) $$\Psi_{\mathrm{seq}}\left(t\right)=\sum_{i=1}^{45}\Psi_{\mathrm{soil}}\left(z_{i},t\right)\cdot {\hat{U}}_{P}\left(z_{i},t\right).$$

Note that Ψ_seq_ is defined for the pot as a whole and does not depend on z. ${\hat{U}}_{P}$ in Equation (4) is equivalent to the standard sink fraction used by Couvreur et al. (2012) which is the profile of RWU rates under conditions of uniformly distributed soil water potential. Note that ${\hat{U}}_{P}$ is normalized and thus $\sum_{i=1}^{45}{\hat{U}}_{P}\left(z_{i},t\right)=1$. From Equation (4), we see that the Ψ_soil_ in layers with a higher root conductance (roughly corresponding to a higher root length) contributes more to Ψ_seq_ than the Ψ_soil_ in layers with a lower root conductance. In the case of a uniform Ψ_soil_ distribution, Ψ_seq_ and Ψ_soil_ are equal. In the following paragraphs, we explain how we obtained Ψ_soil_ and ${\hat{U}}_{P}$ to calculate Ψ_seq_.

The water retention curve of the soil substrate (Supplemental Figure S7) used in our experiments was measured with an evaporation method (Peters and Durner, 2008) and the HYPROP setup (METER Group, Munich, Germany). We used the Brooks–Corey parameters determined by fitting the soil water retention curve to derive the soil matric potential (*h*) and the soil conductivity (*K*_Soil_) in each layer:

(5) $$h\left(\theta ,\;z_{i}\right)=\alpha^{-1}{\left(\frac{\theta (z_{i})-\theta_{r}}{\theta_{s}-\theta_{r}}\right)}^{-\frac{1}{\lambda_{b}}}$$

(6) $$K_{\mathrm{soil}}\left(\theta ,\;z_{i}\right)=K_{\mathrm{sat}}\cdot {\left(\alpha \cdot h\left(\theta ,\;z_{i}\right)\right)}^{\tau }.$$

The Brooks–Corey parameters are saturated water content θ_s_, residual water content θ_r_, air entry pressure head $\alpha^{-1}$ (cm) which depends on the soil pore sizes, a dimensionless pore size index λ_b_, the saturated soil conductivity *K*_sat_ and τ which is derived from λ_b_ with $\tau =-2-3\cdot \lambda_{b}$. The values of all Brooks–Corey parameters of our soil substrate are given in Table 4. The local Ψ_soil_ was obtained by adding a gravity component to *h*:

(7) $$\Psi_{\mathrm{soil}}\left(\theta ,\;z_{i}\right)=\left(h\left(\theta ,\;z_{i}\right)-z_{i}\right)\cdot 9.8\cdot 10^{-5}\frac{\mathrm{MPa}}{\mathrm{cm}}.$$

**Table 4:** Brooks–Corey parameters of our soil substrate derived from the water retention curve

Parameter	Value
*θ*_s_	0.4 mL cm^−3^
*θ*_r_	0.0 mL cm^−3^
*α*	0.072 cm^−1^
*λ*_b_	0.43
*K*_sat_	113.7 cm d^−1^
*τ*	−3.29

A detailed description on deriving ${\hat{U}}_{P}$ from SWaP measurements is provided by van Dusschoten et al. (2020) which we will summarize here. The soil water depletion rate $\frac{\partial \theta }{\partial t}$ in each layer is the sum of the RWU rate (RWU)and redistributive soil water flow (rSWF) between adjacent layers:

(8) $$\frac{\partial \theta \left(z_{i},t\right)}{\partial t}=\;\mathrm{RWU}\left(z_{i},\;t\right)+\mathrm{rSWF}\left(z_{i},\;t\right).$$

Analogously to the model of Couvreur et al. (2012), we write RWU as the sum of *U*_P_ and a second term which corrects *U*_P_ for vertical gradients in the soil water potential. We call this second term soil-driven RWU redistribution (*U*_S_):

(9) $${\mathrm{RWU}\left(z_{i},\;t\right)=U}_{P}\left(z_{i},\;t\right)+\;U_{S}\left(z_{i},\;t\right),\;$$

with $U_{P}\left(z_{i},\;t\right)=\frac{{\hat{U}}_{P}\left(z_{i}\right)\cdot \;U_{\mathrm{tot}}\left(t\right)\;}{V}$, total pot volume *V*, and $U_{\mathrm{tot}}\left(t\right)=\sum_{i=1}^{45}\frac{\partial \theta \left(z_{i},t\right)}{\partial t}$. Note that *U*_S_ is negative in layers with Ψ_soil_ < Ψ_seq_ (lower water uptake rates compared with conditions of uniform Ψ_soil_) and positive in layers with Ψ_soil_ > Ψ_seq_ (higher water uptake rates compared with conditions of uniform Ψ_soil_). In total, the negative corrections just compensate the positive ones and thus $\sum_{i=1}^{45}U_{S}\left(z_{i},t\right)=0$. Similarly, also rSWF sums up to zero: $\sum_{i=1}^{45}\mathrm{rSWF}\left(z_{i},t\right)=0$. We summarize *U*_S_ and rSWF as soil water redistribution through soil and roots ($S_{r}$) and reformulate Equation (8):

(10) $$\frac{\partial \theta \left(z_{i},t\right)}{\partial t}={\hat{U}}_{P}\left(z_{i}\right)\cdot \frac{U_{\mathrm{tot}}\left(t\right)}{V}+S_{r}\left(z_{i},t\right),$$

$\frac{\partial \theta \left(z_{i},t\right)}{\partial t}$ and *U*_tot_ (*t*) can be directly derived from the SWaP measurements of *θ* (*z_i_*, *t*). The two terms in Equation (10) react differently to a change in light intensity. While *U*_tot_, and thus the first term, responds within minutes, the response of the soil-driven water flow, *S*_R_, is much slower. Therefore, the variation of $\frac{\partial \theta \left(z_{i},\;t\right)}{\partial t}$, induced by the fluctuating light intensity, can be solely attributed to a variation of the first term in Equation (10). Given these considerations, we can derive ${\hat{U}}_{P}\left(z_{i}\right)$ as slope of the linear relation between $\frac{\partial \theta \left(z_{i},\;t\right)}{\partial t}$ and *U*_tot_ (*t*) in each layer. SWaP data from 7.00 a.m. (midpoint of first daily high light level) to 7.00 p.m. (midpoint of last daily high light level) were used for the regression. The entire process of deriving ${\hat{U}}_{P}\left(z_{i}\right)$ from the SWaP measurements is exemplified by one faba bean plant in Supplemental Figure S8. This analysis was performed for each day of the soil drying experiment separately, resulting in one ${\hat{U}}_{P}$ profile per day. In contrast, Ψ_soil_ was determined every 15 min. For the calculation of Ψ_seq_, we therefore linearly interpolated the ${\hat{U}}_{P}$ profiles with the measured profiles fixed at the center of the regression period at 12.00 a.m. each day. Given both, Ψ_soil_ and ${\hat{U}}_{P}$ in each layer and point in time, we derived Ψ_seq_ according to Equation (4).

### Measurements of Ψ_leaf_ and stomatal conductance

We continuously measured Ψ_leaf_ on the youngest fully developed leaf using a thermocouple leaf psychrometer (ICT International, Armidale, Australia). Before attaching the psychrometer, the leaf cuticle was carefully removed with abrasive paper. For synchronization with SWaP measurements, Ψ_leaf_ was recorded every 15 min. Stomatal conductance (*g*_s_) was measured using a portable LiCor 6400 photosynthesis system (LiCOR Inc., Lincoln, Nebraska, USA) with a transparent cuvette head. The cuvette was attached to a leaf adjacent to the leaf used for the psychrometer measurements. Since we measured multiple plants at once with only one LiCor 6400, measurements of *g*_s_ were not performed continuously but only once per light period at least 45 min after a change in light intensity to allow *g*_s_ to reach steady state. Stomatal conductance was measured at least during four light periods a day and for six replicates per species only.

### Calculating the water potential at the root surface

Carminati and Javaux (2020) recently suggested that with proceeding soil drying strong water depletion zones around the roots occur leading to a drop of the soil water potential at the root surface. This local drop of the soil water potential was claimed to drive stomatal closure during drought. The Ψ_soil_ that we derived here with the SWaP is based on a measure of the average water content in a soil layer. Thus, Ψ_soil_ better approximates the water potential in the bulk soil than at the root surface. To estimate how strong *K*_SL_ is affected by the hydraulic pathway from bulk soil to root surface, the water potential at the root surface, Ψ_sr_ needs to be known. To calculate Ψ_sr_, we followed the approach described Carminati and Javaux (2020) which is summarized in Supplemental Methods S1 and derived in more detail by Abdalla et al. (2022):

(11) $$\psi_{sr}={\left[\frac{\Phi_{\mathrm{sr}}\cdot (\tau +1)\cdot \alpha^{-\tau }}{K_{\mathrm{sat}}}\right]}^{\frac{1}{\tau +1}},$$

$\Phi_{\mathrm{sr}}$ is the matrix flux potential at the root surface which is given by

(12) $$\Phi_{\mathrm{sr}}=\Phi_{\mathrm{bulk}}-\frac{U_{\mathrm{tot}}}{2\pi r_{0}L}\left(\frac{r_{0}}{2}-r_{0}r_{b}^{2}\frac{\ln\left(r_{b}/r_{0}\right)}{r_{b}^{2}-{r_{0}}^{2}}\right),$$

where *L* is the root length, *r*_0_ is root radius, and *r*_b_ is the radius defining the start of the bulk soil which is approximated by $r_{b}=\sqrt{\frac{V}{\pi L}}$ with the soil volume *V*. $\Phi_{\mathrm{bulk}}$ is the matrix flux potential in the bulk soil which can be derived from Ψ_soil_ analogously to Equation (11):

(13) $$\Phi_{\mathrm{bulk}}=\frac{\alpha^{\tau }}{\left(\tau +1\right)}{\cdot K}_{\mathrm{sat}}\cdot {\psi_{\mathrm{soil}}}^{\tau +1}.$$

According to Equation (12), the difference between $\Phi_{\mathrm{sr}}$ and $\Phi_{\mathrm{bulk}}$ and thus the difference between Ψ_sr_ and Ψ_soil_ increases with increasing water uptake rate per unit root length. For our analysis, we calculated the equivalent water potential at the root surface (Ψ_seq_, _sr_) by using Ψ_seq_ in Equation (13) instead of Ψ_soil_. With the calculated Ψ_seq_, _sr_, we could derive the hydraulic conductance from bulk soil to the root surface (*K*_SR_) and from the root surface to the leaf (*K*_RL_) separately:

(14) $$K_{\mathrm{SR}}=\frac{U_{\mathrm{tot}}}{\psi_{\mathrm{seq}}-\psi_{\mathrm{seq},\;\mathrm{sr}}}$$

and

(15) $$K_{\mathrm{RL}}=\frac{U_{\mathrm{tot}}}{\psi_{\mathrm{seq},\;\mathrm{sr}}-\psi_{\mathrm{leaf}}}.$$

*K*_SR_ and *K*_RL_ are related to *K*_SL_ as follows:

(16) $$\frac{1}{K_{\mathrm{SL}}}=\frac{1}{K_{\mathrm{SR}}}+\frac{1}{K_{\mathrm{RL}}}.$$

### Statistical analyses

We used Mann–Whitney *U* tests (Mann and Whitney, 1947) to test for statistical differences in several parameters between faba bean and maize. The Mann–Whitney *U* test is a nonparametric test for two independent samples, testing the null hypothesis that each of two randomly selected values from two different samples have the same probability of being greater than the other value. Mann–Whitney *U* test were performed using the SciPy package (Virtanen et al., 2020) in Python.

## Supplemental data

The following materials are available in the online version of this article.

**Supplemental Methods S1.**

**Supplemental Figure S1.** Relation between *K*_SL_ (black) and Ψ_seq_ and *g*_s_ (orange) and Ψ_seq_ for all measured faba bean plants separately.

**Supplemental Figure S2.** Relation between *K*_SL_ (black) and Ψ_seq_ and *g*_s_ (orange) and Ψ_seq_ for all measured maize plants separately.

**Supplemental Figure S3.** Daily trend of *K*_SL_.

**Supplemental Figure S4.** Water potential at the root surface (Ψ_seq, sr_) as a function of the bulk soil water potential (Ψ_seq_) for different scenarios.

**Supplemental Figure S5.** Distribution of root length (*L*) and RWU rates (${\hat{U}}_{P}$).

**Supplemental Figure S6.** Recovery of *U*_tot_ and Ψ_leaf_ upon rewatering for different faba bean plants.

**Supplemental Figure S7.** Water retention curve of the soil substrate used in the experiments.

**Supplemental Figure S8.** Determination of ${\hat{U}}_{P}$ profiles from the SWaP data on the local soil water depletion rate $\frac{\partial \theta \left(z_{i},t\right)}{\partial t}$ and the total root water uptake rate *U*_tot_ (*t*).

## Supplementary Material

kiac422_Supplementary_Data

## Notes

Y.M., J.A.P., H.P., and D.v.D. designed the research. Y.M. and D.v.D. performed the research. Y.M. analyzed the data and wrote the manuscript. All authors contributed to the discussion and revision of the manuscript.

The author responsible for distribution of materials integral to the findings presented in this article in accordance with the policy described in the Instructions for Authors (https://academic.oup.com/plphys/pages/general-instructions) is: Dagmar van Dusschoten (d.van.dusschoten@fz-juelich.de).
