# Supplementary material for: Stomatal conductance tracks soil-to-leaf hydraulic conductance in faba bean and maize during soil drying
Source: Plant Physiol. 2022 Sep 13;190(4):2279–94. doi: 10.1093/plphys/kiac422 (PMC9706430; doi:10.1093/plphys/kiac422)
Supplement: kiac422_Supplementary_Data [file kiac422_supplementary_data.pdf]

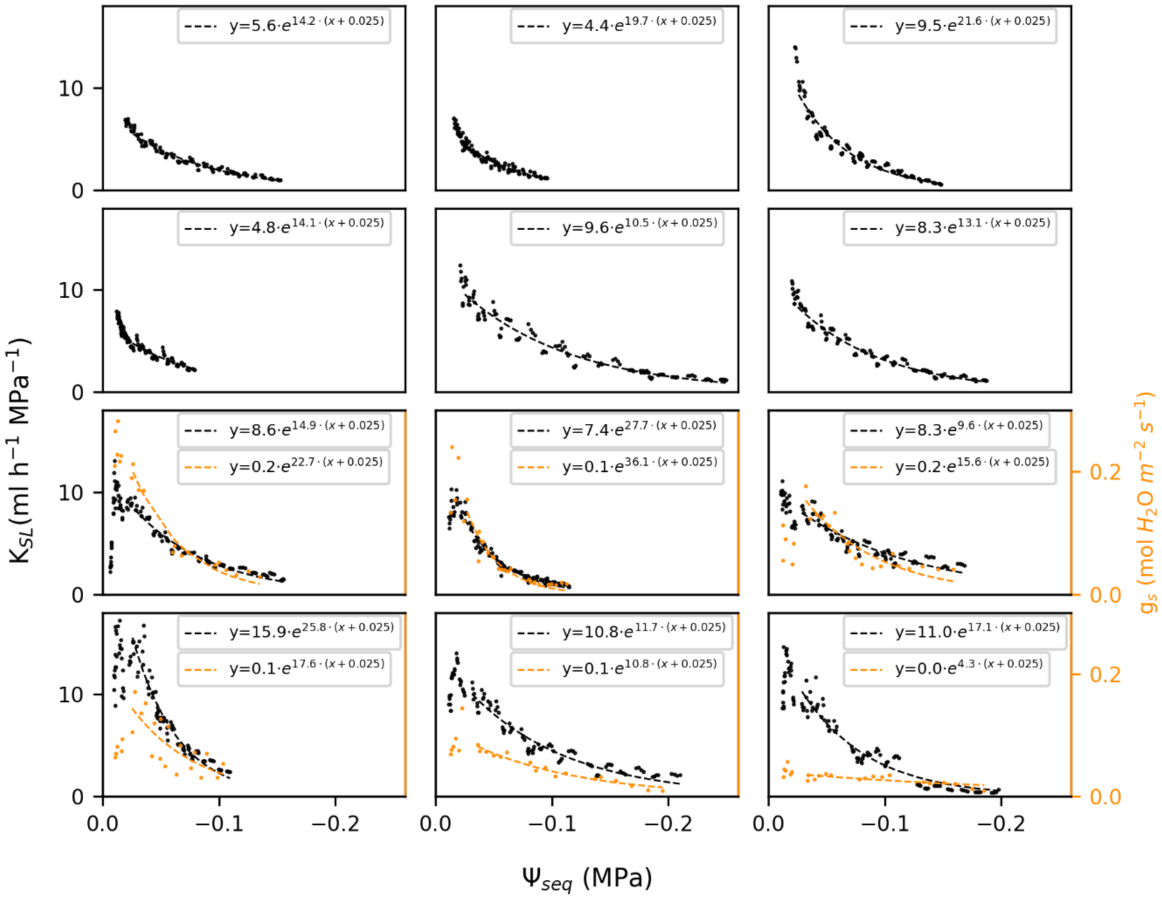

Supplemental Fig. S1 Relation between K_{SL} (black) and Ψ_{seq} and g_s (orange) and Ψ_{seq} for all measured faba bean plants separately. Dashed lines show an exponential fit of the data starting at a Ψ_{seq} of -0.025 MPa.

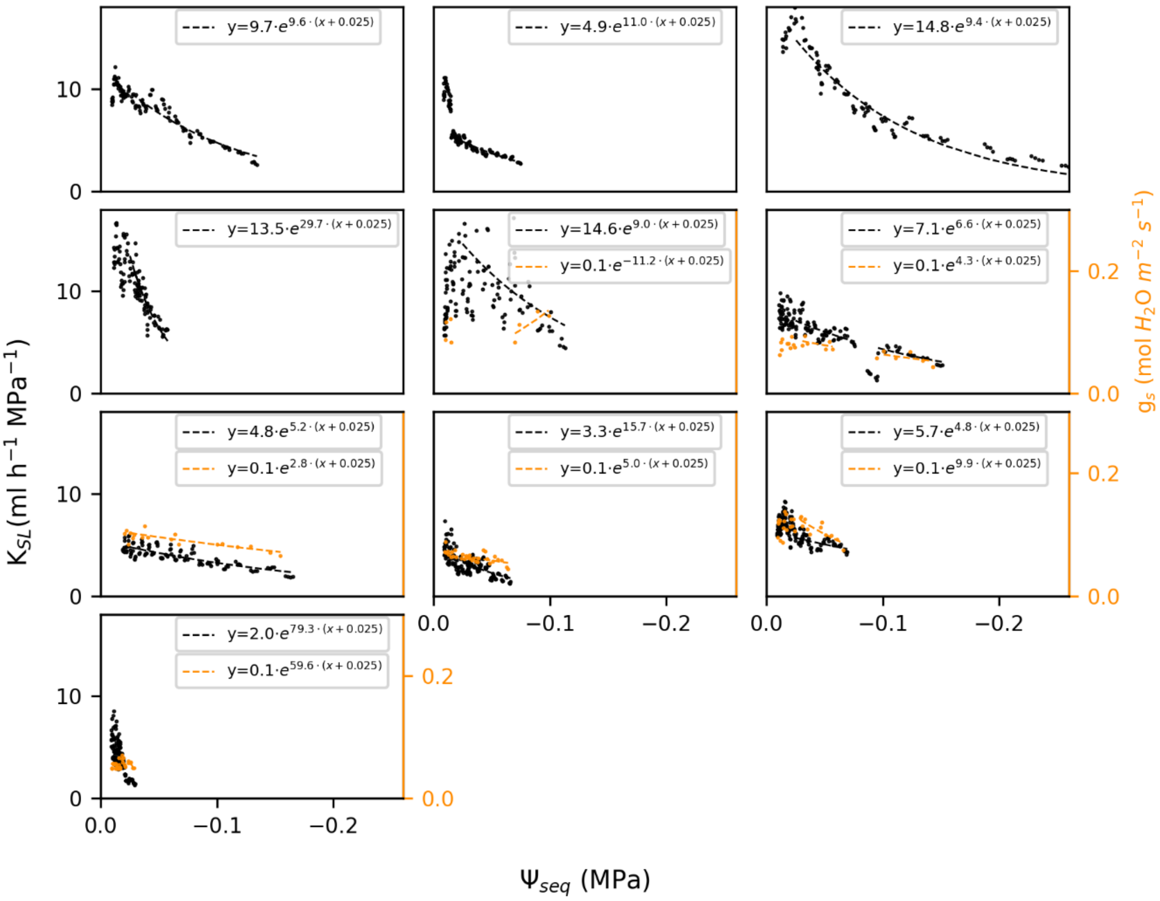

Supplemental Fig. S2 Relation between K_{SL} (black) and Ψ_{seq} and g_s (orange) and Ψ_{seq} for all measured maize plants separately. Dashed lines show an exponential fit of the data starting at a Ψ_{seq} of -0.025 MPa.

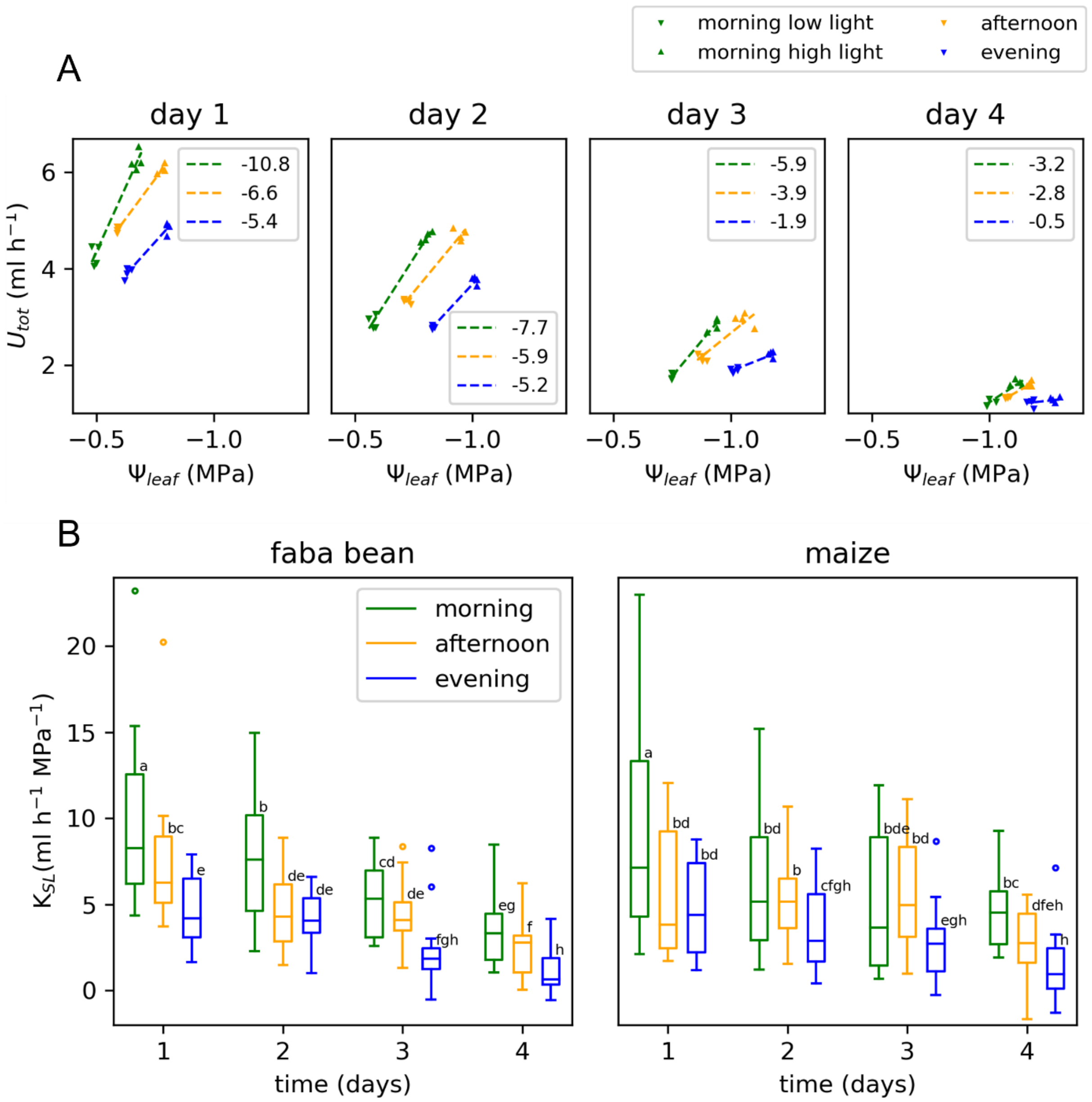

Supplemental Fig. S3 Daily trend of K_{SL}. A) Relation between U_{tot} and Ψ_{leaf} at three different time intervals each day, exemplary shown for one faba bean plant. Four data points per low light period (downward triangles) and high light period (upward triangles) in the morning (green), afternoon (orange) and evening (blue) are shown. K_{SL} is given as slope of the relation between U_{tot} and Ψ_{leaf} which we derived by linearly fitting the data from each time interval separately (values in ml h^{-1} MPa^{-1} in the figure legends). B) Boxplots of K_{SL} derived as shown in A) for all replicates. Characteristics of the boxplots are similar to Fig. 2 A, C, E. Significant differences (P-value of a paired Student's t-test <0.05) between the different time points are denoted by different letters.

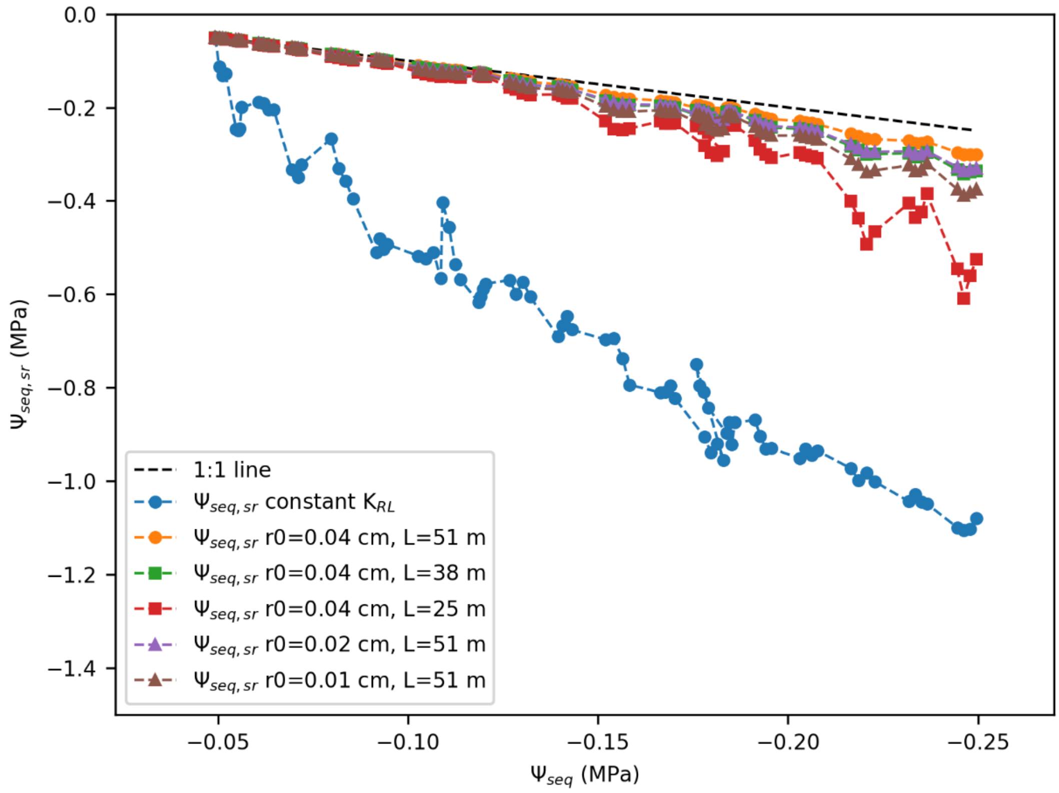

Supplemental Fig. S4 Water potential at the root surface ($\Psi_{seq,\ sr}$) as a function of the bulk soil water potential (Ψ_{seq}) for different scenarios. Data are from one exemplary faba bean plant. The blue curve is the theoretical $\Psi_{seq,\ sr}$ for the scenario of a constant K_{RL}, meaning that the measured decline in K_{SL} had solely occurred in the soil. It was calculated using eq. 1 with a constant, initial K_{SL}. The orange curve is the estimated $\Psi_{seq,\ sr}$ using the model and 100% of both, the measured total root length (L) and the measured average root radius (r0). The other curves are the model estimations of $\Psi_{seq,\ sr}$ if only a reduced fraction of either L (green and red) or r0 (purple and brown) are considered. The green and purple curves are very similar making the green curve hardly visible.

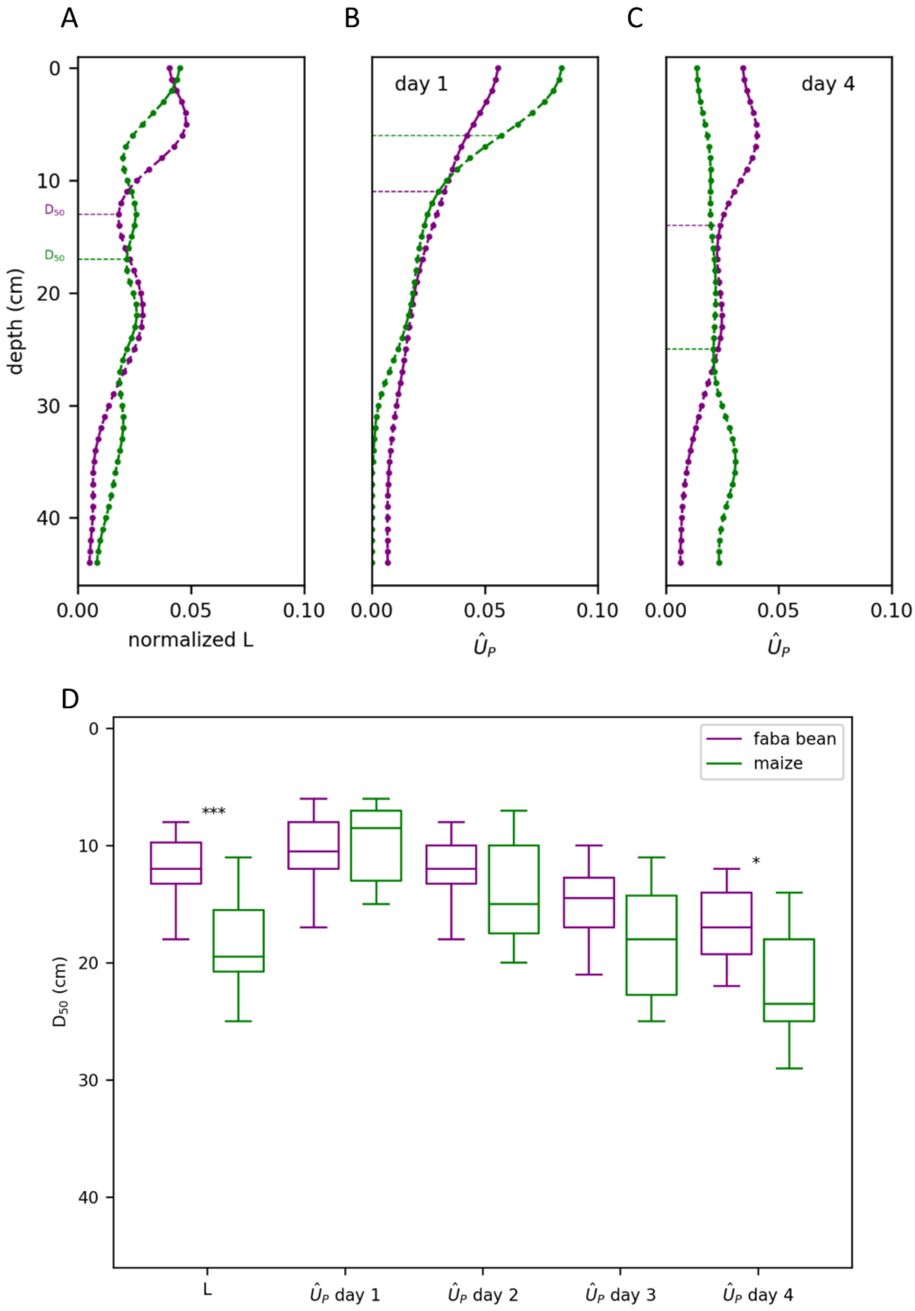

Supplemental Fig. S5 Distribution of root length (L) and root water uptake rates ($\widehat{U}_P$). Exemplary data of L (A), and $\widehat{U}_P$ at the first (B) and last day (C) of measurement for one faba bean and one maize plant. Horizontal dashed lines in A-C indicate the depth (D_50) at which 50% of L or $\widehat{U}_P$ were reached. D) Boxplots of D_50 for L and $\widehat{U}_P$ during the four days of measurement. Asterisks indicates significant differences between faba bean and maize, determined with a Mann-Whitney U test. P-values below 0.05 are indicated by *, below 0.01 by ** and below 0.005 by ***. Characteristics of the boxplots are similar to Fig. 2 A, C, E.

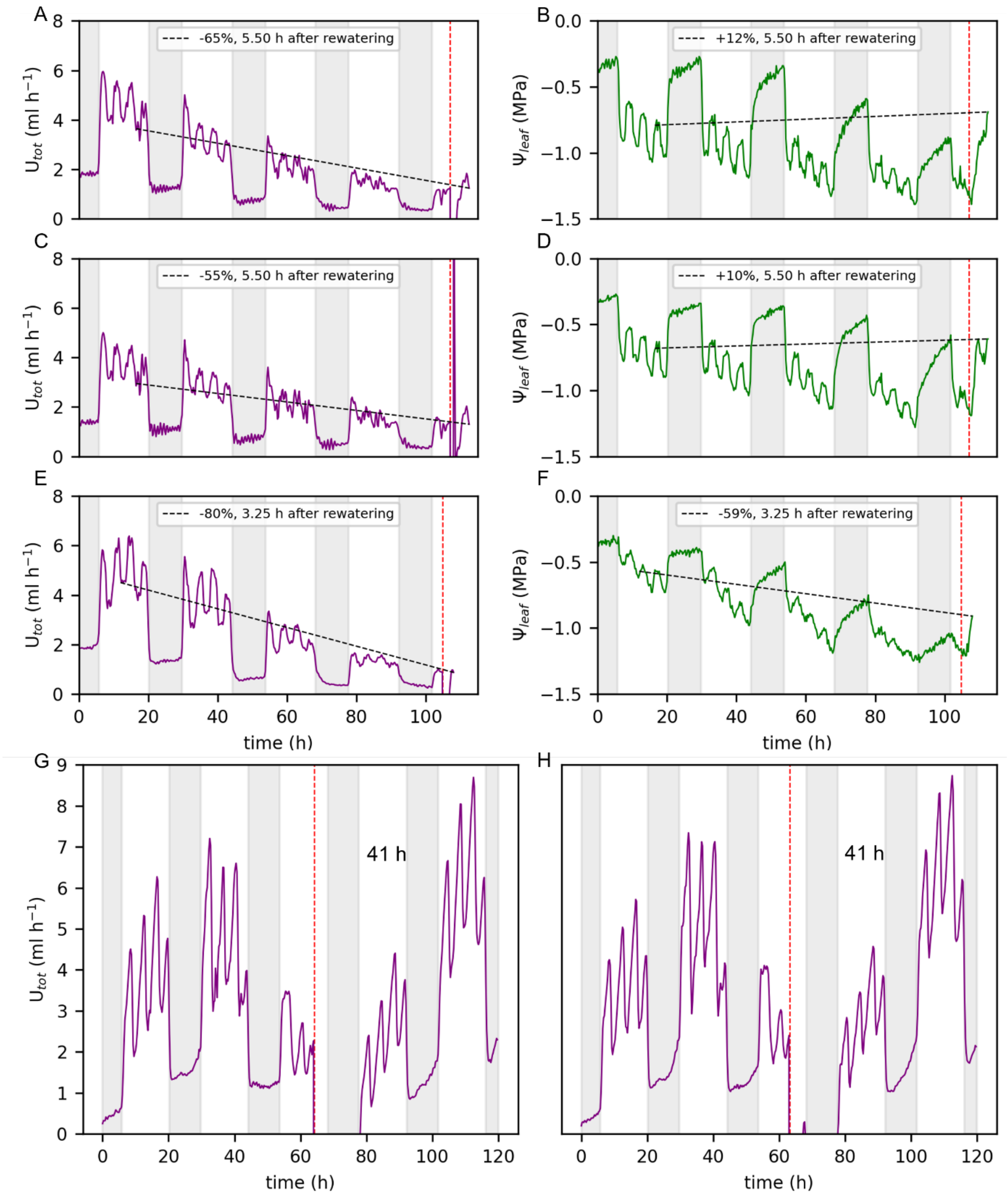

Supplemental Fig. S6 Recovery of U_{tot} and Ψ_{leaf} upon rewatering for different faba bean plants. Data of U_{tot} (A, C, E) and Ψ_{leaf} (B, D, F) are from three faba bean plants, measured in the main experiment of this study. Black dashed lines connect the last measured point after rewatering with the corresponding point (same time of day) at the first day of measurement. The percentage difference between these two points is shown in the figure legends together with the time passed between rewatering and the last measured point. (G and H) Longer recovery period of U_{tot} after rewatering for two six weeks old faba bean plants from a separate experiment without measurements of Ψ_{leaf}. In both plants it took

around 41 hours of recovery for U_{tot} to reach the maximum values from before rewatering. Nights are indicated by the shaded areas.

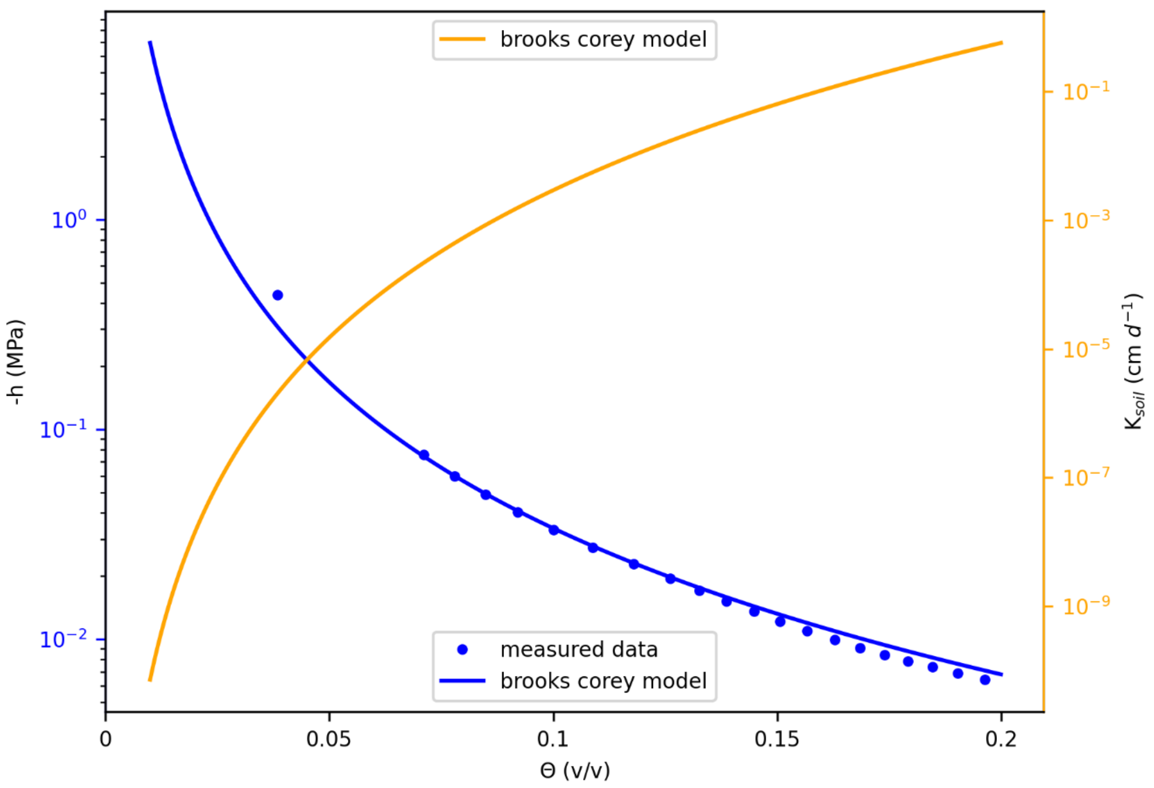

Supplemental Fig. S7 Water retention curve of the soil substrate used in the experiments. Blue data points are the measured, negative matric potentials h (left y-axis). Blue line is the fitted matric potential using the Brooks-Corey model (eq. 5). Orange line is the Brooks-Corey model of the soil hydraulic conductivity (K_{soil}, eq. 6) (right y-axis). The obtained values of the Brooks-Corey parameters are given in Table 4. X-axis is the relevant range of volumetric soil water content Θ in our study.

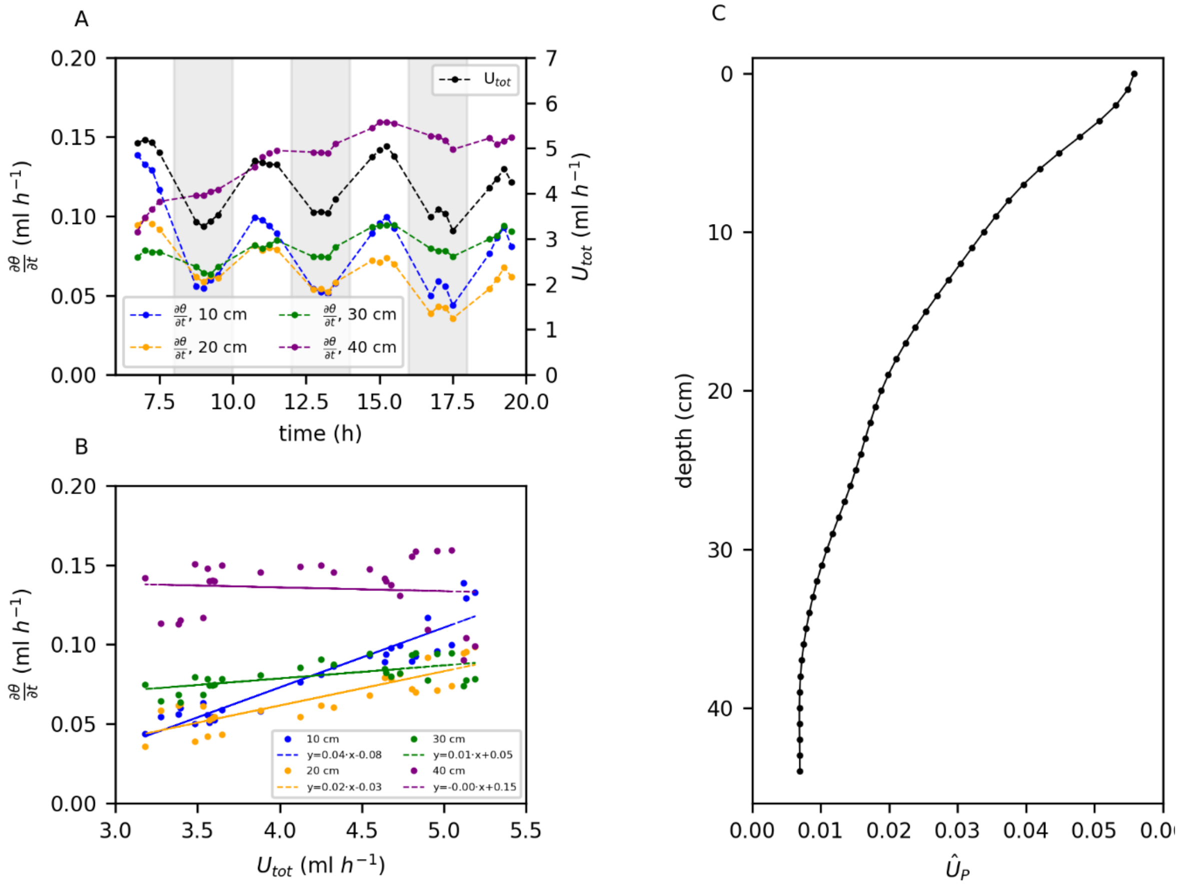

Supplemental Fig. S8 Determination of $\widehat{U}_P$ profiles from the SWaP data on the local soil water depletion rate $\frac{\partial\theta(z_i,t)}{\partial t}$ and the total root water uptake rate U_{tot} (t). A) Response of $\frac{\partial\theta(z_i,t)}{\partial t}$ in different depths (different colors, left y-axis) and U_{tot} (black, right y-axis) to the fluctuating light intensity. White areas indicate a high light period, grey areas a low light period. B) Determination of $\widehat{U}_P$ in each soil layer as slope of the linear relation between $\frac{\partial\theta(z_i,t)}{\partial t}$ and U_{tot} according to eq. 8. C) Resulting $\widehat{U}_P$ profile of one exemplary faba bean plant at the first day of measurement. Note that this entire process was necessary to ultimately derive Ψ_{seq} as a precise measure for the level of soil drying sensed by the plant.

Supplemental methods S1

A Kirchhoff transformation of the soil hydraulic conductivity (K_{Soil}) yields the matrix flux potential (ϕ) at a certain location x:

$$\Phi_x = \int_{-\infty}^{\psi_x} K_{soil}(\psi) d\psi \qquad [1]$$

For a Brooks-Corey soil, this writes:

$$\Phi_x = \int_{-\infty}^{\psi_x} K_{sat} \cdot (\alpha \cdot \psi)^{\tau} d\psi \qquad [2]$$

with the saturated soil hydraulic conductivity K_{sat}, the air entry pressure head α^{-1} and a fitting parameter τ. Solving eq 2 results in a relation between the matrix flux potential and the water potential.

$$\Phi_x = \frac{\alpha^{\tau}}{(\tau+1)} \cdot K_{soil} \cdot {\psi_x}^{\tau+1} \qquad [3]$$

Solving eq 3 for Ψ at the root surface gives

$$\psi_{sr} = \left[\frac{\Phi_{sr} \cdot (\tau+1) \cdot \alpha^{-\tau}}{K_{sat}}\right]^{\frac{1}{\tau+1}} \quad [4]$$

Additionally, the Kirchhoff transformation can be used to solve the radial 1D Richards equation for water flow from bulk soil towards the root surface which yields a relation between the matrix flux potential at the root surface (ϕ_{sr}) and in the bulk soil (ϕ_{bulk}):

$$\Phi_{sr} = \Phi_{bulk} - \frac{\mathrm{U}_{tot}}{2\pi r_0 L}\left(\frac{r_0}{2} - r_0 r_b^2 \frac{ln(r_b/r_0)}{r_b^2 - {r_0}^2}\right) \qquad [5]$$

with root length L, root radius r_0 and the radius defining the start of the bulk soil r_b which is approximated by $r_b = \sqrt{\frac{V}{\pi L}}$ with the soil volume V. Given eq. 3-5, Ψ_{sr} was derived as follows:

1. Calculating ϕ_{bulk} using eq. 3 and the measured bulk soil water potential together with the Brooks-Corey parameters of our soil
2. Calculating ϕ_{sr} with the derived ϕ_{bulk} according to eq. 5
3. Calculating Ψ_{sr} with the derived ϕ_{sr} and the Brooks-Corey parameters according to eq. 3.
